# The A2b Adenosine Receptor Modulates Glucose Homeostasis and Obesity

**DOI:** 10.1371/journal.pone.0040584

**Published:** 2012-07-25

**Authors:** Hillary Johnston-Cox, Milka Koupenova, Dan Yang, Barbara Corkey, Noyan Gokce, Melissa G. Farb, Nathan LeBrasseur, Katya Ravid

**Affiliations:** 1 Departments of Medicine, Boston University School of Medicine, Boston, Massachusetts, United States of America; 2 Department Biochemistry, Boston University School of Medicine, Boston, Massachusetts, United States of America; 3 Whitaker Cardiovascular Institute, Boston University School of Medicine, Boston, Massachusetts, United States of America; 4 Evans Center for Interdisciplinary Biomedical Research, Boston University School of Medicine, Boston, Massachusetts, United States of America; University of Colorado Denver, United States of America

## Abstract

**Background:**

High fat diet and its induced changes in glucose homeostasis, inflammation and obesity continue to be an epidemic in developed countries. The A2b adenosine receptor (A2bAR) is known to regulate inflammation. We used a diet-induced obesity murine knockout model to investigate the role of this receptor in mediating metabolic homeostasis, and correlated our findings in obese patient samples.

**Methodology/Principal Findings:**

Administration of high fat, high cholesterol diet (HFD) for sixteen weeks vastly upregulated the expression of the A2bAR in control mice, while A2bAR knockout (KO) mice under this diet developed greater obesity and hallmarks of type 2 diabetes (T2D), assessed by delayed glucose clearance and augmented insulin levels compared to matching control mice. We identified a novel link between the expression of A2bAR, insulin receptor substrate 2 (IRS-2), and insulin signaling, determined by Western blotting for IRS-2 and tissue Akt phosphorylation. The latter is impaired in tissues of A2bAR KO mice, along with a greater inflammatory state. Additional mechanisms involved include A2bAR regulation of SREBP-1 expression, a repressor of IRS-2. Importantly, pharmacological activation of the A2bAR by injection of the A2bAR ligand BAY 60-6583 for four weeks post HFD restores IRS-2 levels, and ameliorates T2D. Finally, in obese human subjects A2bAR expression correlates strongly with IRS-2 expression.

**Conclusions/Significance:**

Our study identified the A2bAR as a significant regulator of HFD-induced hallmarks of T2D, thereby pointing to its therapeutic potential.

## Introduction

Glucose homeostasis is maintained through a delicate balance of exogenous dietary intake, endogenous hepatic release, and peripheral clearance by the adipose tissue and muscle. In obesity or T2D, there is decreased ability of insulin to clear circulating glucose, defined as insulin resistance [1]. Insulin resistance and obesity are two important risk factors that contribute to the pathogenesis of T2D. Poor glycemic control, central obesity, and dyslipidemia contribute to a prothrombotic and proinflammatory state, heightening the risk for an adverse cardiovascular event [2], [3], [4]. On the other hand, inflammatory cytokines, such as TNF-α, are reported to contribute to T2D through activation of inflammatory pathways, including Jun N-terminal kinase (JNK) and inhibitor of κB kinase (IKKβ) (reviewed in [5]), both of which stimulate transcription of inflammatory pathway genes and phosphorylate insulin receptor substrate (IRS) and insulin receptors, interfering with normal insulin signaling [6], [7].

Adenosine, a purine nucleoside, is an important metabolite that is released from cells following insult or inflammation (reviewed in [8]). Adenosine in the extracellular space can bind to four different subtypes of G-protein coupled receptors, classified as adenylyl cyclase inhibitory (A1 and A3) or adenylyl cyclase activating (A2b and A2a) [9], [10]. Adenosine, acting through the A2-type receptors, has been implicated in mediating inhibition of the release of inflammatory cytokines at baseline, post-injury and post-bacterial challenge (reviewed in [11]). In addition to the mediation of inflammation, adenosine and its receptors have been linked to the regulation of glucose clearance [12], [13].

Previous studies have shown that the A2bAR plays a significant role in controlling inflammation, with A2bAR KO mice displaying a pro-inflammatory phenotype (16). Considering that HFD is a known inducer of an inflammatory profile [14], and given the implication of inflammation in the development of insulin resistance, we developed a diet-induced obesity model to explore the role of the A2bAR in the context of glucose clearance and tissue insulin sensitivity. Results obtained show that the contribution of the A2bAR to the control of glucose homeostasis is different under HFD than under regular diet [15]. We report a significant upregulation of the A2bAR by HFD, and indicate the A2bAR as protective against HFD-induced insulin resistance. Our investigations also point to mechanisms involved.

## Results

### A2bAR Null Mice under High Fat Diet (HFD) are Obese with Impaired Glucose and Insulin Homeostasis

**Figure 1:**
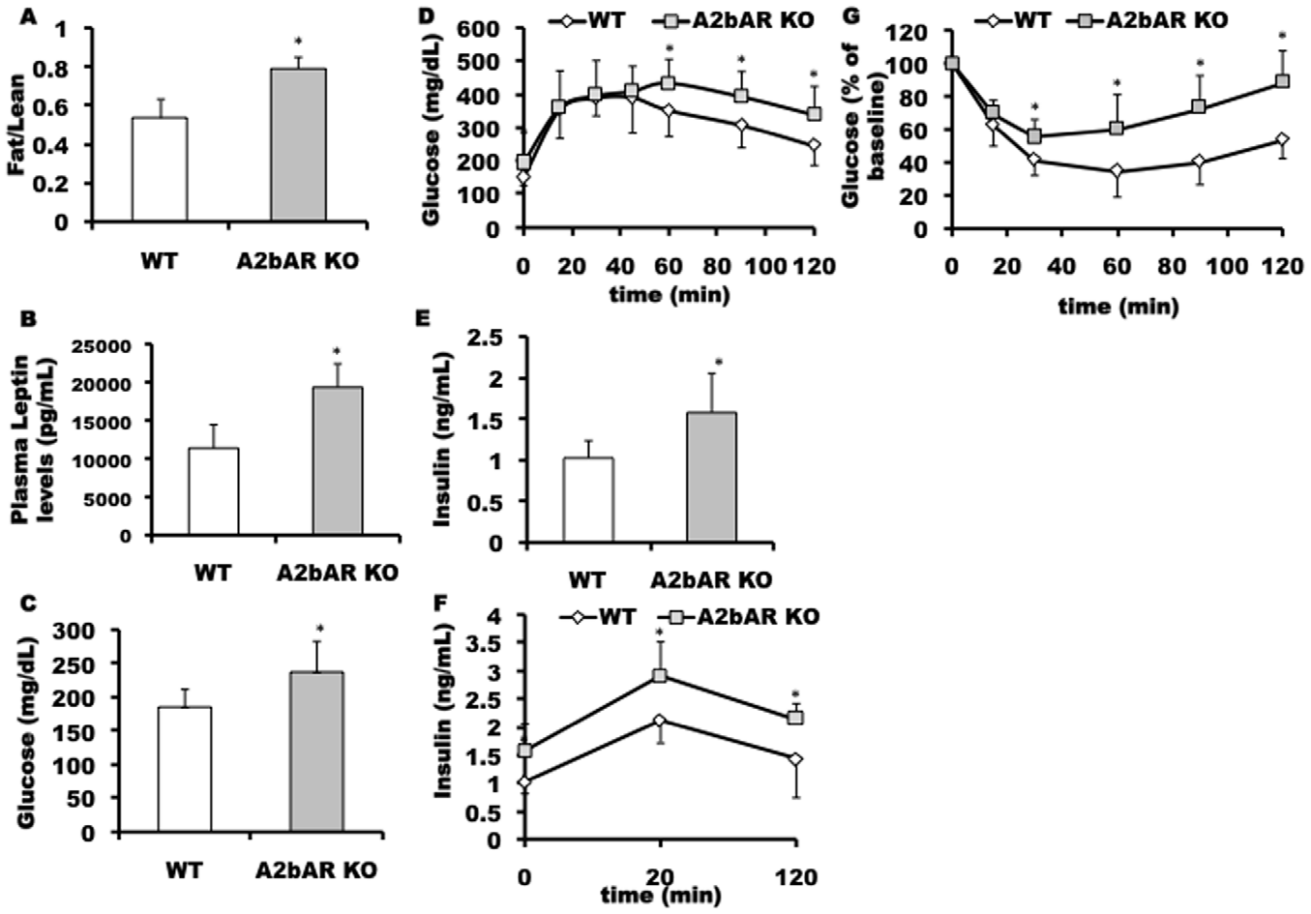
Effect of A2bAR on body composition and on glucose and insulin homeostasis post HFD. Matching control (WT) and A2bAR KO male mice (see methods) at 16 weeks post HFD were subjected to **A.** fat to lean mass ratio measurements (n = 12/group), p-value = 0.0007; and to **B.** measurement of effect of A2bAR elimination on the plasma levels of the lipokine, leptin (n = 6), p-value = 0.0033. **C.** Blood glucose levels 16 hours post starvation (n = 12/group), p-value = 0.0016. **D.** Glucose clearance in the blood post glucose overload (n = 12/group), p-values = 0.0007 (0 min); 0.0407 (60 min); 0.0229 (90 min); 0.0128 (120 min). **E.** Insulin levels (n = 8/group), p-value = 0.0195. **F.** Insulin clearance in the plasma post glucose overload (n = 8/group), p-values = 0.0412 (0 min); 0.0354 (20 min); 0.0451 (120 min). **G.** Glucose clearance in the plasma post insulin overload (n = 8/group), graphed as percentage of baseline, p-values = 0.0062 (30 min); 0.0085 (60 min); 0.0008 (90 min); 0.0002×10̂(−1) (120 min).

Given the contribution of HFD-induced inflammation to the development of T2D (reviewed in [16]), and the role of the A2bAR in controlling inflammation, we sought to characterize the metabolic outcome of HFD in absence of the A2bAR. In the course of HFD administration, wild type (WT) and A2bAR KO mice gained weight in a similar fashion (Fig. S1A), and displayed no difference in food consumption (Fig. S1B). Consistently with the growth pattern and food intake, there were no differences observed in metabolic rate measured by oxygen consumption (Fig. S1C), or heat production (Fig. S1D). Looking at the respiratory exchange ratio (RER), measuring differences in fuel source for energy supply, such as carbohydrates (RER of 1.0) vs. fat (RER of 0.7) or mixed (RER of 0.85), we did not observe a significant difference between WT control and A2bAR KO mice (Fig. S1E). Intriguingly, however, post HFD mice lacking the A2bAR had higher fat to lean mass ratio relative to WT control (Fig. 1A). Consistent with the increase in fat to lean ratio, the A2bAR KO mouse post HFD had elevated plasma leptin. Plasma leptin levels are highly correlated with adipose tissue mass [17], (Fig. 1B).

One of the potential hallmarks of T2D is obesity (reviewed in [5], [18]). Thus, we sought to examine the role of A2bAR in glucose and insulin homeostasis under HFD. Interestingly, A2bAR KO mice had higher baseline glucose levels (Fig. 1C), and decreased glucose clearance evaluated by glucose tolerance test (GTT), compared to WT mice under the same diet (Fig. 1D), confirmed by quantification of the area under the curve (AUC) (Fig. S2A). In addition, baseline insulin levels (Fig. 1E) and insulin levels post glucose overload were higher in the A2bAR KO mice (Fig. 1F), suggesting inefficiency in glucose clearance and increase in insulin resistance. A2bAR KO mice also displayed an impaired insulin tolerance test (ITT) (Fig. 1G), further demonstrative of insulin resistance. In accordance with insulin resistance, A2bAR KO mice also demonstrated a compromised pyruvate tolerance test (Fig. S2B). Furthermore, primary islets isolated from the pancreas of these mice showed hyper-secretion as a result of HFD and high glucose challenge, as well as on chow diet, (Fig. S3A, S3B), suggestive of an endogenous effect of A2bAR on pancreatic secretion of insulin. This latter finding is consistent with studies showing that pharmacological inhibition of the A2bAR in mice leads to insulin hypersecretion [19], which could explain a previous report of augmented glucose clearance in A2bAR KO mice under chow diet [15].

### Tissues of A2bAR KO Mice under HFD Display Compromised Insulin Signaling and an Altered Inflammatory Profile

**Figure 2:**
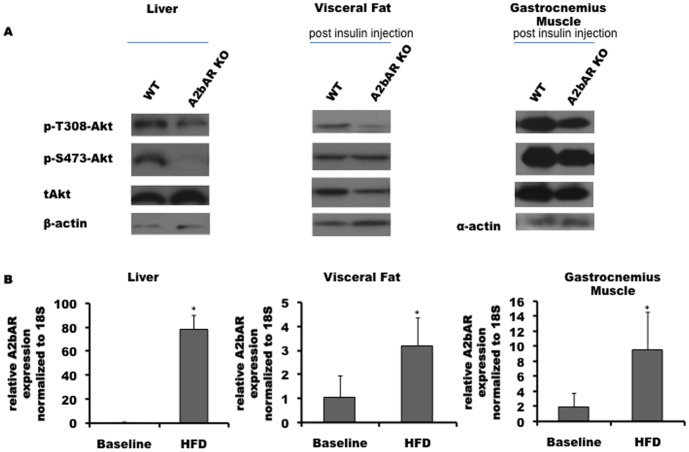
Effect of A2bAR elimination on tissue insulin signaling post HFD. Western blot analysis of different insulin responsive tissues; one representative WT and A2bAR KO pair is shown. **A.** phospho-Thr308 Akt (60 kDa), phospho-Ser473 Akt (60 kDa), total Akt (60 kDa) and β-actin (43 kDa) in liver at base line, and in visceral fat tissue and gastrocnemius tissue 15 minutes post-insulin injection (1 U/kg Humulin R ). **B.** Tissue A2bAR mRNA levels were measured by qPCR. Twelve-week-old WT mice (C57BL/6J) were analyzed and compared to similar mice subjected to additional 16 weeks of HFD. The expression of the receptor is relative to the baseline value, measured in 12 week old mice before the 16 weeks diet (denoted as HFD), and normalized to 18 S rRNA (denoted as 18 S), p-value = 0.0087 for liver; p-value = 0.0073 for visceral fat; p-value = 0.0009 for gastrocnemious muscle.

Intriguingly, under HFD A2bAR KO mice showed impaired insulin signaling in the liver, visceral fat, and gastrocnemius muscle (Fig. 2A). This was assessed by measuring the level of serine/threonine phosphorylated Akt, which was compromised in the liver at baseline and in other tissues post insulin challenge. Examples of analyses of additional WT and A2bAR KO pairs can be found in Supplementary Materials (Fig S4). Examination of expression of endogenous A2bAR showed a vast upregulation in the liver following HFD (Fig. 2B), and a modest trend of upregulated A2bAR expression in the visceral fat and gastrocnemius muscle (Fig. 2B). The limited expression of the A2bAR in fat and muscle suggests that the impaired insulin signaling in these tissues in absence of the receptor is also due to systemic effects. In addition, A2bAR expression is upregulated with age in the liver, visceral fat and gastrocnemius muscle (Figure S5A, S5B, S5C), albeit not to the same extent as seen with HFD.

Systemic and tissue specific changes in inflammatory cytokines are well known to contribute to insulin resistance (reviewed in [20]). As reported in [21], A2bAR KO mice under HFD exhibit an increased inflammatory profile as well, evidenced by elevated plasma levels of TNF-α and monocyte chemotactic protein-1 (MCP-1) (Fig. S6A). A2bAR KO fat tissue also displays augmented TNF-α, compared to control mice under the same HFD (Fig. S6B). Histological analysis of visceral fat showed a modest increase in crown structures in the A2bAR KO mice compared to control, with no significant change in fat cell size (Fig. S6C). The crown structures are indicative of an active inflammatory state [5].

### Livers of A2bAR KO Mice have Reduced Levels of IRS-2

**Figure 3:**
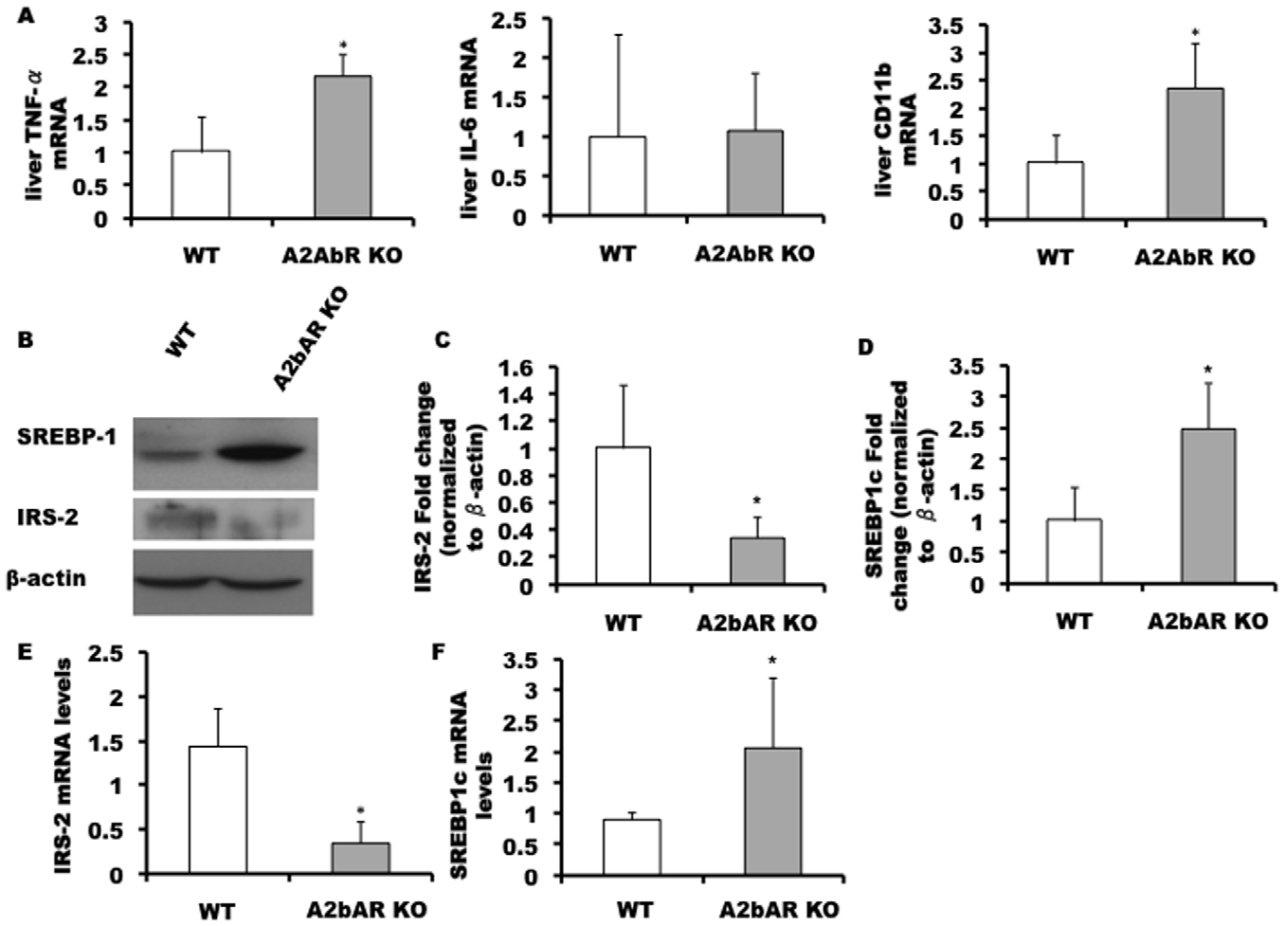
A2bAR elimination causes elevated hepatic inflammatory parameters and a decrease in IRS-2 levels. Livers isolated from mice 16 weeks post HFD were analyzed for **A.** TNF-α, IL-6 and CD-11b mRNA levels, p-value = 0.0413 (TNF-α); p-value = 0.05 (CD11b). **B.** mature SREBP-1 (68 kDa) and IRS-2 (185 kDa) by Western blot analysis, using β-actin as loading control (representative of 4 experiments). **C.** Densitometry-quantitation of IRS-2 data from B., was done using ImageJ, p-value = 0.0429. **D.** Densitometry-quantitation of SREBP-1 data from panel B, using ImageJ, p-value = 0.0171. **E.** mRNA levels of IRS-2 determined by qPCR (normalized to 18 s rRNA), p-value = 0.0070. **F.** mRNA levels of SREBP-1 determined by qPCR (normalized to 18 s rRNA), p-value = 0.0030.

Livers of A2bAR KO mice display elevated levels of TNF-α, IL-6 and CD11b (marker of activated macrophages), compared to control mice under HFD (Fig. 3A). TNF-α has been shown to promote impaired insulin signaling through downregulation of the level of insulin receptor substrate 2 (IRS-2) [22]. In a recent study we showed that cyclic AMP (cAMP) via A2bAR signaling on an Apolipoprotein E (ApoE) null background controls the level of liver SREBP-1 [21], which is an established transcriptional repressor of IRS-2 expression [23], [24]. Here, we show that elimination of A2bAR on normal ApoE background also results in increased SREBP-1 protein (Fig. 3B,3D) and mRNA (Fig. 3F) levels in the liver, and to a significant decrease in IRS-2 protein and mRNA (Fig. 3B, 3C, 3E respectively). Analyses of additional WT and A2bAR KO pairs can be found in supplemental material (Fig S7). Mechanistically, this is highly relevant, since IRS-2 KO mice display T2D associated with compromised insulin signaling in the liver, but not restricted to this tissue [25], [26].

In the liver, SREBP-1 is also known to regulate the expression of enzymes related to liver lipid synthesis [27], as we confirmed in the context of A2bAR signaling in a mouse model of atherosclerosis [21]. In accordance with elevated SREBP-1, livers of the A2bAR KO mice with normal ApoE under HFD demonstrate steatosis (Fig. S8A), with increased triglyceride (TG) and cholesterol content (Fig. S8B, S8C, respectively). Plasma TG and cholesterol levels are also elevated in the A2bAR KO mice (Fig. S8D, S8E, respectively). An augmented lipid profile is a known contributor to T2D as well via influences on altered insulin secretion [28], [29]. In addition, several studies have demonstrated an association between elevated lipid profiles and increased abdominal fat (e.g., [30], [31]). Diets high in saturated fat lead to a positive energy balance, which results in a consequent increase in adipose mass [32]. Thus, the hyperlipidemia in the A2bAR KO mouse could also contribute to the observed altered body composition post HFD.

### Pharmacological Activation of the A2bAR Ameliorates HFD-induced Impaired Glucose Tolerance, Insulin Signaling and Chronic Inflammation

**Figure 4:**
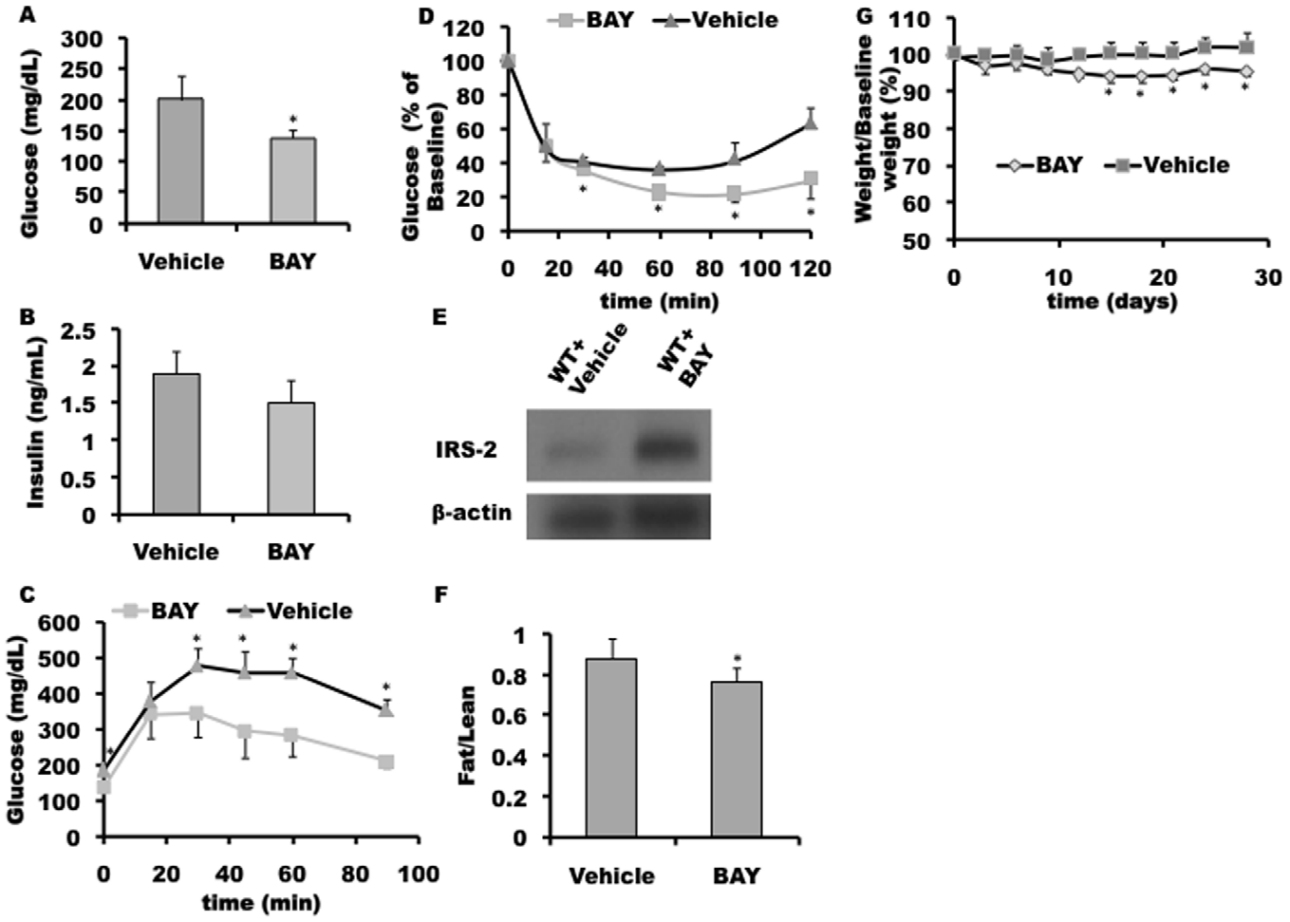
A2bAR activation ameliorates a diabetic profile. Glucose parameters were measured in WT mice 20 weeks post HFD (32 weeks of age), in the last 4 weeks (of the 20) administration of A2bAR specific agonist BAY 60-6583 (denoted as BAY) or Vehicle (denoted as Vehicle) was introduced, following a regime described in Methods. The following parameters were measured: **A.** Blood glucose levels (n = 12/group), p-value = 0.0178. **B.** Insulin levels (n = 8/group). **C.** Glucose clearance in the blood post glucose overload (n = 12/group), p-value = 0.0178 (0 min); 0.0198 (30 min); 0.0163 (45 min); 0.0061 (60 min); 0.0346 (90 min). **D.** Glucose clearance in the plasma as a result of insulin overload (n = 8/group) 6 hours post starvation, graphed as percentage of baseline, p-values = 0.0085 (30 min); 0.0005 (60 min); 0.0248 (90 min); 0.0176 (120 min). **E.** Western blot analysis of hepatic IRS-2 levels post-Bay 60-6583 injection. **F.** Fat to Lean Mass Ratio 4 weeks post-Bay 60-6583 administration, p-value = 0.0227. **G.** Percent weight gain with 4 weeks BAY treatment, p-values = 0.0026 (day 15); 0.0061 (day 18); 0.0078 (day 21); 0.0017 (day 24); 0.0065 (day 28), respectively.

To explore the potential therapeutic effect of the A2bAR on ameliorating a T2D phenotype, wild type (WT) mice (C57BL/6J) were injected intraperitoneally with the A2bAR specific agonist, BAY 60-6583 [33], or vehicle for 4 weeks post 16 weeks HFD. Mice injected with BAY 60-6583 had lower plasma glucose and insulin levels, compared to vehicle-injected mice (Fig. 4A, 4B). In addition, chronic activation of the A2bAR with BAY 60-6583 improved glucose clearance and peripheral tissue insulin sensitivity, demonstrated by GTT and ITT (Fig. 4C, 4D), again compared to mice injected with vehicle. Importantly, systemic BAY 60-6583 injection restored liver IRS-2 levels (Fig. 4E). Analyses of additional WT and A2bAR KO pairs can be found in supplemental material (Fig S9). Levels of TNF-α, which marks acute inflammation, were not different in the two groups (Fig. S10A), but BAY 60-6583 led to reduced levels of IL-6 (Fig. S10B), a marker of chronic inflammation. Indeed, IL-6 has been associated with T2D in both mice and human studies [15]. Additionally, agonism of the A2bAR led to a decrease in fat to lean mass ratio (Fig. 4F) as well as a slight but significant weight loss (Fig. 4G). Administration of BAY 60-6583 to A2bAR KO mice had no influence on glucose and insulin tolerance tests, fat to lean mass ratio or weight, supporting the conclusion that the glucose-lowering effect of BAY 60-6583 in the WT mice is due to a specific effect on the A2bAR (Fig. S11A, S11B, S11C, S11D). These results point to the therapeutic potential of this ligand, as well as support future development of additional A2bAR selective agonists for management of glucose clearance and tissue insulin sensitivity in the aspect of HFD-induced T2D.

### Analysis of Human Adipose Tissues for Inflammation, A2bAR and IRS-2 Expression

**Figure 5:**
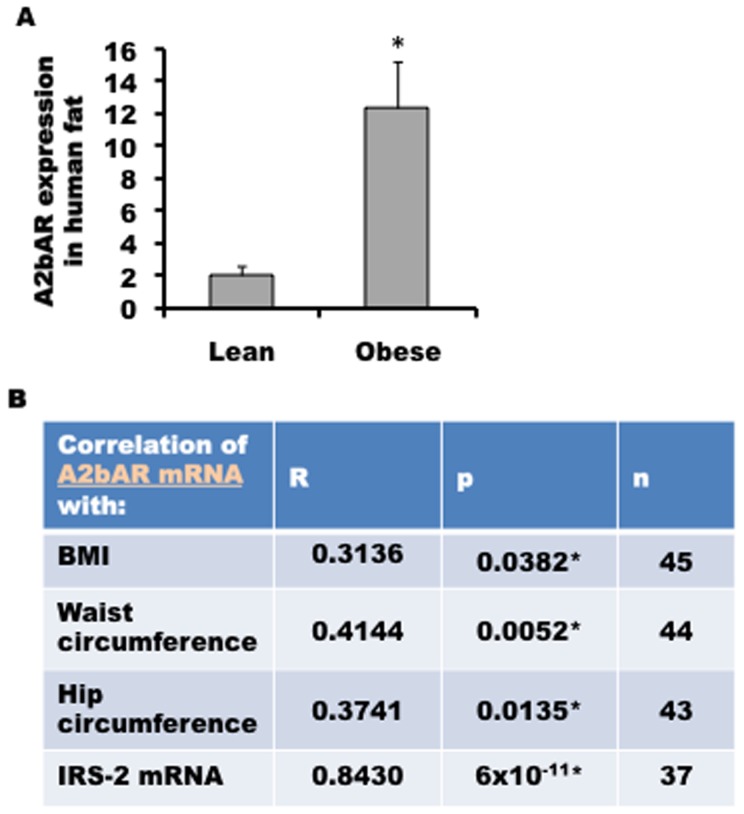
Correlations between the expression of A2bAR and IRS-2 in human samples. **A.** Subcutaneous fat obtained from 19 lean individuals with BMI<25 (8 males and 11 females) or 26 obese individuals with BMI>36 (7 males and 19 females) was collected (see Methods) and used for RNA extraction and qPCR analysis normalized to 18s RNA, p-value = 0.0016. **B.** The Spearman correlation coefficient (R) and p-value (p) were calculated with GraphPad Prism 5 software. P-values for each are listed in the table. Abbreviations are as follows: body mass index (BMI), Insulin receptor substrate 2 (IRS-2). Table S1 lists the mean values for each parameter for the number of people analyzed.

To gain insight on the relevance of our findings in humans, we analyzed subcutaneous fat obtained from a total of 45 individuals characterized as follows: 19 lean individuals with BMI<25 (8 males and 11 females); 26 obese individuals with BMI >36 (7 males and 19 females). There were no samples from individuals with 25<BMI>36 (see Table S1). As shown in Figure 5A, A2bAR expression is higher in the samples derived from obese individuals, compared to lean, in accordance with the mouse data. We also measured A2bAR and IRS-2 mRNA expression, and calculated the correlation between A2bAR mRNA expression and parameters of obesity or IRS-2 mRNA using the Spearman correlation coefficient (R). Noted is the positive correlation between A2bAR expression and obesity (e.g. waist circumference), and particularly a remarkable correlation (R = 0.843; p = 6×10^−11^) between A2bAR and IRS-2 mRNA expression (Figure 5B).

## Discussion

T2D is one of the major risk factors for cardiovascular disease [34], [35]. Adenosine receptors have been studied in the context of insulin homeostasis and glucose metabolism [19], [36], [37], lipolysis, development of hepatic steatosis, and reverse cholesterol transport (e.g., [38], [39]). Our current study identifies a novel role for the A2bAR in the context of T2D, including insulin and glucose homeostasis, chronic inflammation and obesity under HFD.

A2bAR KO mice fed with high fat, high cholesterol diet display altered glucose clearance and insulin secretion and elevated fat to lean mass ratio, compared to WT mice, suggestive of a T2D compensatory phase. Tissue insulin resistance was evident in the A2bAR KO post HFD, noted by alterations in phosphorylation of Akt, an important downstream signaling component of the insulin pathway. In accordance with recent studies in our lab exploring the role of the A2bAR in the context of atherosclerosis and lipid metabolism on an ApoE null background [21], we determined here that the A2bAR on normal ApoE background also regulates hepatic SREBP-1 as well as plasma lipids post HFD challenge. SREBP-1 not only controls the lipid profile via its effect on enzymes involved in lipid synthesis [40], but it also regulates the expression of IRS-2 [23]. Additionally, IRS-2 levels are also known to be increased by cAMP [41] and hepatocyte levels of cAMP are lower upon A2bAR elimination [21]. Indeed, here we show that livers of A2bAR null mice under HFD have significantly reduced levels of IRS-2 compared to control mice, which is expected to result in impaired insulin signaling [24] as confirmed in our system as well.

A central inquiry at hand is whether hyperglycemia in A2bAR KO mice under HFD is a result of an autonomous regulation of pancreatic ß-cells by the A2bAR and/or a result of systemic influences on the pancreas, stemming from A2bAR-induced changes in other tissues, including liver upregulation of triglycerides and cholesterol. This is a relevant inquiry, since insulin resistance can cause hyperglycemia, and hyperinsulinemia, which in turn results in resistance. We propose that both processes contribute to T2D in A2bAR KO mice. HFD leads to vast upregulation of A2bAR in the liver and elimination of A2bAR results in hyperlipidemia, while the latter has been shown to acutely trigger ß-cells to hyper-secrete insulin (e.g., [42], [43]). This raises the possibility that changes in the liver A2bAR affect pancreatic insulin secretion. On the other hand, we (Fig. S3A, S3B), and others [19] found that genetic or pharmacological ablation of the A2bAR leads to increased insulin secretion from isolated pancreatic islets. The mechanism of effect of pancreatic A2bAR on insulin secretion is yet to be explored.

The impaired insulin signaling in non-hepatic tissues of A2bAR KO mice is thought to be mediated primarily by systemic effects, suggested by the slight upregulation of A2bAR with HFD seen in the adipose and muscle tissue relative to robust upregulation noted in the liver. Hepatic A2bAR regulates the lipid profile [21], and expression of the A2bAR in sites such as the liver or macrophages [44] controls the level of inflammatory cytokines. High levels of TNF-α and IL-6 have been reported in multiple diabetic and insulin resistant states [45], [46]. TNF-α disrupts insulin signaling through both inhibitory phosphorylation and reduction in total levels of IRS-1/IRS-2 and downstream signaling ([47], reviewed in [48]). The systemic and tissue pro-inflammatory profile in the A2bAR KO could contribute to the observed downregulated IRS-2, and impaired Akt signaling, and to the peripheral tissue insulin resistance. Future studies could explore the contribution of inflammation induced by the macrophage A2bAR, to the insulin resistance seen in the A2bAR KO. Chronic inflammation associated with obesity is thought to be an important factor in the pathogenesis of T2D [49], [50]. Recent studies have identified sensors of “danger signals”, such as the Nlrp3 inflammasome, to contribute to obesity-related inflammation and insulin resistance [51]. A2bAR is known to regulate inflammatory cytokines as TNF-alpha and IL-6 [11], and perhaps other danger signals, such as ceramides or free fatty acids could potentially synergize or integrate with A2bAR signals to activate the inflammasome; exploring this possibility would constitute a new line of investigation.

Our findings in subcutaneous fat from obese patients suggest that the A2bAR should be further explored as a marker of IRS-2 expression and of obesity, as well as considered as a therapeutic target. Indeed, by using a selective A2bAR agonist in vivo in mice, we showed that the A2bAR is protective against HFD-induced T2D phenotypes. A recent study [15] reported that A2bAR ablation improves glucose homeostasis and clearance in mice fed a regular chow diet, while we find that under HFD, this receptor deletion has an opposite effect. Under both diets, A2bAR ablation leads to pancreatic insulin hypersecretion (Fig. S3A, S3B) [19]. Under regular diet, this might lead to a rapid glucose clearance, as reported in [15], as there is no impairment of insulin signaling. Under HFD, A2bAR KO mice, despite elevated insulin levels, demonstrate impaired glucose clearance. This is indicative of tissue insulin resistance in A2bAR KO mice. This suggests that in this case, hyperinsulimia alone is not sufficient to cause insulin resistance. On the other hand, following HFD, liver A2bAR is vastly upregulated, resulting in maintenance of adequate levels of IRS-2 and reduced lipids, which are expected to guard against HFD-induced insulin resistance.

In summary, our study elucidates a role for the A2bAR in maintenance of glucose and insulin homeostasis, modulation of inflammatory mediated events, and lipid metabolism. We establish a role for A2bAR in the regulation of IRS-2 expression and effects on downstream insulin signaling. In absence of the A2bAR, elevated plasma and tissue inflammatory markers contribute to impaired insulin response, while pharmacological activation of the A2bAR modulates chronic inflammation and restores IRS-2 signaling. The translational value of this receptor is further enhanced with data that shows a positive association between the A2bAR and BMI, parameters of obesity (e.g. waist and hip circumference) and IRS-2 in subcutaneous fat samples obtained from obese patients. It is of interest to study if activation of the A2bAR in type 2 diabetic patients can improve insulin resistance and inflammatory parameters.

## Materials and Methods

### Ethics Section

All mice procedures were approved and in agreement with the guidelines of and approved by the Institutional Animal Care and Use Committee of the Boston University School of Medicine (Protocol AN14064).

For human subject data, all protocols were approved by the Boston Medical Center Institutional Review Board and all subjects provided written informed consent.

### Animal Models and Diets

The A2bAR KO/β-galactosidase knock-in mouse model used in these studies have been generated by our laboratory and previously described [52]. Age-matching WT control male mice were purchased from Jackson Laboratory (cat# 000664) (details are provided in the online supplement, Methods S1) or bred as littermates for confirmatory *experiments.*

### Metabolic Measurements

#### Oxygen consumption, heat production, respiratory exchange ratio (RER)

Indirect calorimetry was performed with mice individually housed with free access to food and water in Oxymax calorimeter cages (Columbus Instruments, Columbus, OH, USA) (details are provided in online data supplement, Methods S1).

#### Body-composition analysis

Body composition was assessed using nuclear magnetic resonance (NMR) measurements (EchoMRI, Echo Medical Systems, Houston, TX, USA), and EchoMRI software (version 2007.08.10) on 28-week-old WT (n = 12) and A2bAR KO (n = 12) mice post 16 weeks HFD (details are provided in online data supplement, Methods S1).

#### In vivo glucose, insulin and pyruvate tolerance tests

Mice were put on HFD for 14 weeks, and starved for 16 hours for glucose tolerance and 6 hours for insulin and pyruvate tolerance tests (details are provided in online data supplement, Methods S1).

#### Plasma analysis for cholesterol and triglycerides

Cholesterol and triglycerides were measured as previously described [21].

#### Pancreatic islet isolation and insulin secretion

Pancreatic islets were isolated and cultured as described in [53] (details are provided in online data supplement, Methods S1).

### Immunohistochemistry

Paraffin sections of visceral fat and liver post HFD were subjected to F4/80 and H&E staining, respectively (details are provided in online data supplement, Methods S1).

### Quantitative Reverse-transcriptase Polymerase Chain Reaction (qPCR)

RNA was isolated from different tissues and subjected to qPCR as described in [21] (details are provided in online data supplement, Methods S1).

### Western Blot Analysis

Protein was extracted from tissues and subjected to western blotting as described previously [52] (details are provided in online data supplement, Methods S1).

### ELISA for Measurement of Cytokine Levels

Following euthanasia, blood was collected by cardiac puncture, plasma was isolated as described in [21], and cytokine levels were measured (details provided in the online data supplement, Methods S1).

### BAY 60-6583 Injection

Mice were injected intraperitoneally for 4 weeks post 16 weeks HFD, every third day with BAY 60-6583 (2 µg/g mouse, dissolved in 100 µl vehicle: 50% PEG-400, 50% water [54]) or with equal volume of vehicle. During BAY injection, mice were still fed a HFD. At 4 weeks post-BAY mice underwent GTT and ITT; at 4 weeks post-BAY injection mice underwent NMR analysis as described above. At completion mice were starved for 16 hours and collected for analysis.

### Human Sample Collection, cDNA Preparation and qPCR

We enrolled adult men and women (age≥18 years; range: 21–56 years old) with different body mass indexes (BMI range 20–51 kg/m^2^) from the Boston Medical Center Nutrition and Weight Management Program. Fat samples were collected from the subcutaneous abdominal fat region by biopsy procedure. mRNA levels of genes were quantified by using qPCR (details on sample collection and procedures are provided in online data supplement).

### Statistics

The data from each experiment is expressed as mean ± standard deviation (SD). Statistical comparison was done using two-tail student t-test assuming equal variance and considered significant when p≤0.05 (*). Area under the curve (AUC) for GTT was generated using GraphPad Prism 5 software. As to human data, expression of A2bAR is graphed as mean ± standard error (SE). Correlation between A2bAR expression and the epidemiological data was done using Spearman correlation coefficient, as described in [55]. All statistical analyses were performed using GraphPad Prism 5 software.

## Supporting Information

Figure S1 **Effect of A2bAR elimination on different metabolic parameters.** Wild type (WT) and matching A2bAR KO (A2bAR KO) mice at 28 weeks of age (16-weeks post HFD) were subjected to different measurements at either fasted or fed conditions as described in methods. **A.** Weight gain normalized to baseline weight (n = 8). **B.** Food intake, measured as a difference between new food and leftovers over the period of one week, per cage and divided by the number of mice in that particular cage. n = 8. **C.** Metabolic rate. **D.** Heat production. **E.** Respiratory exchange ratio. In cases **C–E** data is representative of n = 4/group. All measurements are described under methods.
(TIF)

Figure S2 **Glucose tolerance test area under the curve (AUC) and pyruvate tolerance test. A.** The area under the curve (AUC) was calculated using GraphPad Prism 5 software for plasma glucose levels post-glucose challenge, p-value = 0.0311. **B.** Plasma glucose levels post-pyruvate challenge as described in methods (n = 4/group), p-values = 0.0237 (0 min); 0.0160 (30 min).
(TIF)

Figure S3 **Insulin secretion measured in primary islets.** Measurements as described under methods, were carried out in islets treated with basal (3 mmol/L, denoted as 3 G) glucose and high glucose (12 mmol/L, denoted as 12 G) **A.** at baseline (before HFD) and **B.** 4 weeks post HFD, p-value = 0.0283.
(TIF)

Figure S4 **Effect of A2bAR elimination on tissue insulin signaling post HFD.** Western blot analysis of additional A2bAR KO and WT pairs for Akt signaling in the liver (A) or fat (B) or gastrocnemious muscle (C), with all details as shown in Figure 2.
(TIF)

Figure S5 **A2bAR tissue expression with age and HFD.** Tissue A2bAR mRNA levels in **A.** Liver, **B.** Visceral fat, **C.** Gastrocnemius (Gastroc.) muscle, were measured by qPCR. Twelve-week-old WT mice (C57BL/6J) were analyzed and compared to both similar mice subjected to additional 16 weeks of HFD or an additional 16 weeks of regular chow diet (RD). The expression of the receptor is relative to the baseline value, measured in 12 week old mice before the 16 weeks diet (denoted as HFD or RD), and normalized to 18 S rRNA (denoted as 18 S), p-value = 0.0006, 0.1183×10̂−8 for liver; p-value = 0.0284, 0.1839×10̂−8 for visceral fat; p-value = 0.0011, 0.0009 for gastrocnemious muscle.
(TIF)

Figure S6 **A2bAR elimination causes increase in inflammation and macrophage infiltration.** **A.** TNF-α, IL-6 and MCP-1 levels were measured post 16 weeks of HFD in the plasma, p-value = 0.0007, 0.75116, 0.0392, respectively. The mice were 28 week old upon collection for analysis. **B.** TNF-α, IL-6 and MCP-1 levels were measured post HFD in the visceral fat, p-value = 0.0367. In each group data is representative of n = 10 for plasma and n = 4 for mRNA levels. qPCR data were normalized to 18 S rRNA and presented as A2bAR KO samples relative to WT (set at 1). **C.** Morphology of visceral fat paraffin sections (12 week old mice subjected to 16 weeks of HFD, and collected at 28 weeks of age) stained with F4/80 to detect macrophages, and counterstained with hematoxylin, as described in methods. Arrows point to crown structures (F4/80-positive cells). Data are representative of 5 sections and 3 mice per group. There is a trend towards an increase (but not a statistically significant one) in the number of crown structures in the KO samples compared to WT control. Similarly, analysis of fat cell size using Image J software show no statistical difference in adipocyte cell size in WT and KO samples.
(TIF)

Figure S7 **A2bAR elimination causes elevated SREBP-1 and a decrease in IRS-2 levels.** Western blot analysis of additional A2bAR KO, WT pairs for SREBP-1 and IRS-2 levels, with all details as shown in Figure 3.
(TIF)

Figure S8 **Role of A2bAR in liver lipid homeostasis. A.** Liver morphology post 16 weeks HFD depicted in cryo sections stained with H&E as described in the methods. Noted are the fat droplets in the A2bAR KO samples. **B., C.** Liver cholesterol (n = 6) and triglyceride (n = 6) content in WT and A2bAR KO mice measured as described in Methods. **D., E.** Plasma lipid levels, including triglycerides (TG), p-value = 0.0002 and cholesterol, p-value = 0.0895×10̂(−3), were measured in 28-week-old male mice following 16 weeks of HFD and 16 hours post starvation.
(TIF)

Figure S9 **Effect of A2bAR activation on IRS-2 level.** Shown are additional analyses of effects of A2bAR activation by vehicle (V) or BAY 60-6583 (BAY) on IRS-2 levels in wild type mice, with all details as shown in Figure 4E.
(TIF)

Figure S10 **BAY 60-6583 administration ameliorates chronic inflammation.** All conditions are as described in the legend to Figure 4. Plasma levels of TNF-α (**A**) and IL-6 (**B**), p-value = 0.0138, at Baseline (12 weeks), Pre-treatment (16 weeks HFD), and post-BAY 60-6583 (BAY**)** or Vehicle injection.
(TIF)

Figure S11 **Specificity of BAY 60-6583 in vivo.** Glucose parameters were measured in male A2bAR KO (A2bAR KO) 20 W post High fat diet (26 weeks of age), post-4 weeks administration of A2bAR specific agonist BAY 60-6583 (denoted as BAY) or Vehicle. **A.** Glucose clearance in the blood post glucose overload (n = 5/group), **B.** Glucose clearance in the plasma post insulin overload (n = 5/group), graphed as percentage of baseline. **C.** Fat to lean mass ratio (n = 5/group), **D.** Percent weight gain with 8 weeks BAY 60-6583 treatment (n = 5/group).
(TIF)

Table S1 **Clinical Characteristics.** Values are a mean ± standard deviation. The following abbreviations were used: body mass index (BMI), low density lipoprotein (LDL), high density lipoprotein (HDL); * p-value <0.05; **p-value<0.001.
(TIF)

Methods S1 Supplemental methods are available online labeled as S1 Methods.
(DOC)
