# Supplementary figures and images for: The A2b Adenosine Receptor Modulates Glucose Homeostasis and Obesity
Source: PLoS One. 2012 Jul 25;7(7):e40584. doi: 10.1371/journal.pone.0040584 (PMC3405065; doi:10.1371/journal.pone.0040584)

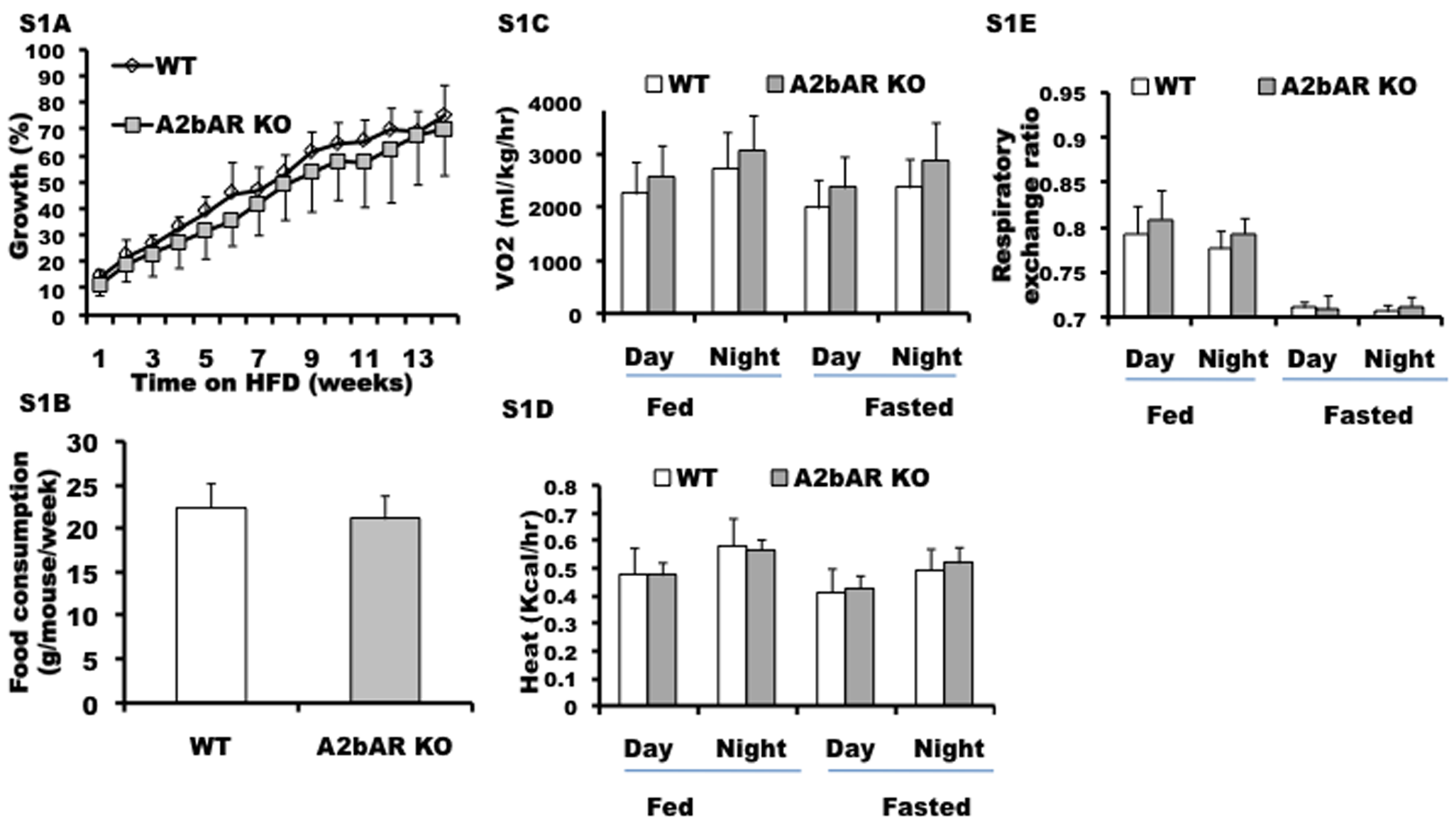

Supplement: Figure S1 — Effect of A2bAR elimination on different metabolic parameters. Wild type (WT) and matching A2bAR KO (A2bAR KO) mice at 28 weeks of age (16-weeks post HFD) were subjected to different measurements at either fasted or fed conditions as described in methods. A. Weight gain normalized to baseline weight (n = 8). B. Food intake, measured as a difference between new food and leftovers over the period of one week, per cage and divided by the number of mice in that particular cage. n = 8. C. Metabolic rate. D. Heat production. E. Respiratory exchange ratio. In cases C–E data is representative of n = 4/group. All measurements are described under methods. (TIF) [file pone.0040584.s001.tif]

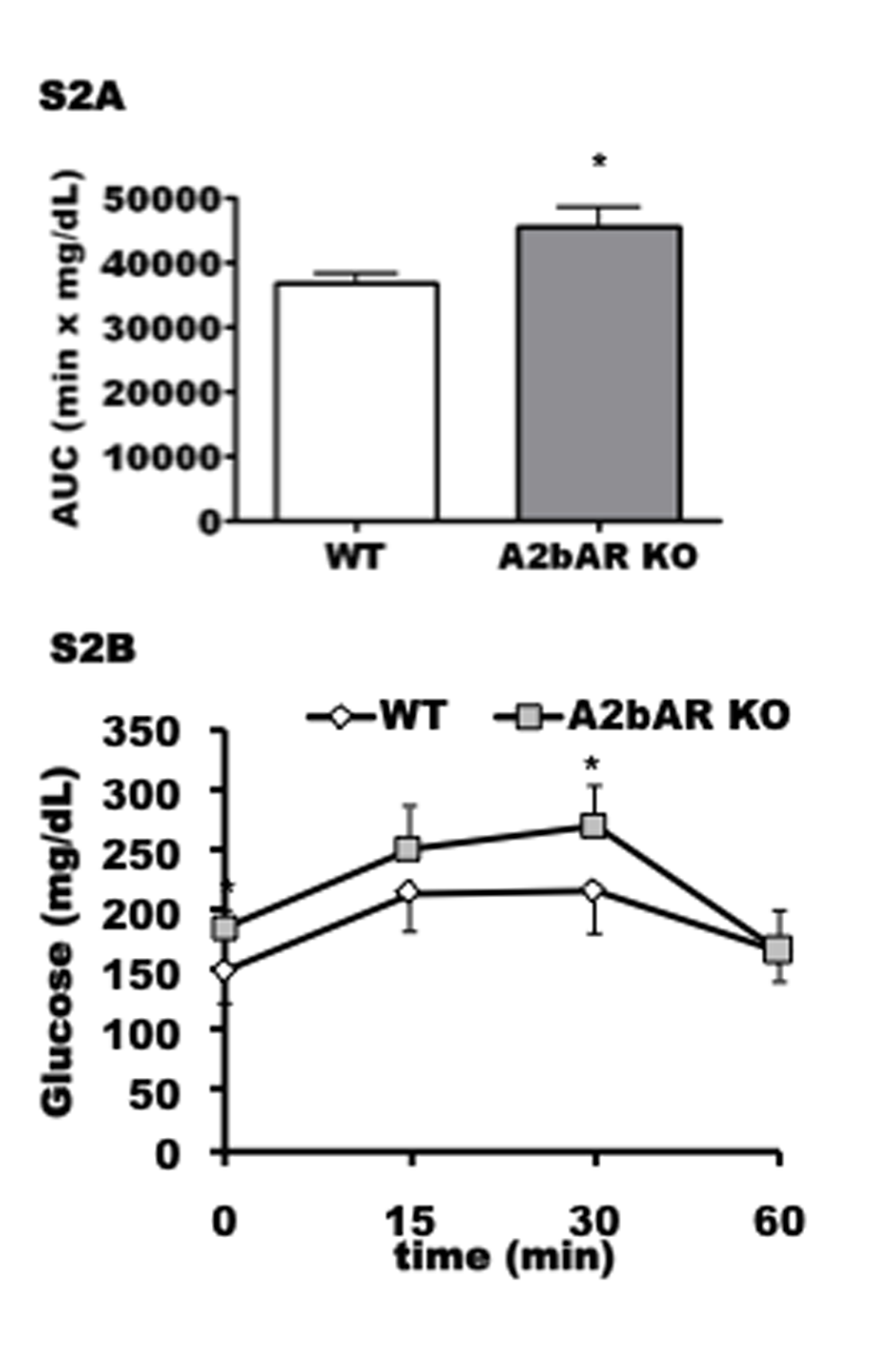

Supplement: Figure S2 — Glucose tolerance test area under the curve (AUC) and pyruvate tolerance test. A. The area under the curve (AUC) was calculated using GraphPad Prism 5 software for plasma glucose levels post-glucose challenge, p-value = 0.0311. B. Plasma glucose levels post-pyruvate challenge as described in methods (n = 4/group), p-values = 0.0237 (0 min); 0.0160 (30 min). (TIF) [file pone.0040584.s002.tif]

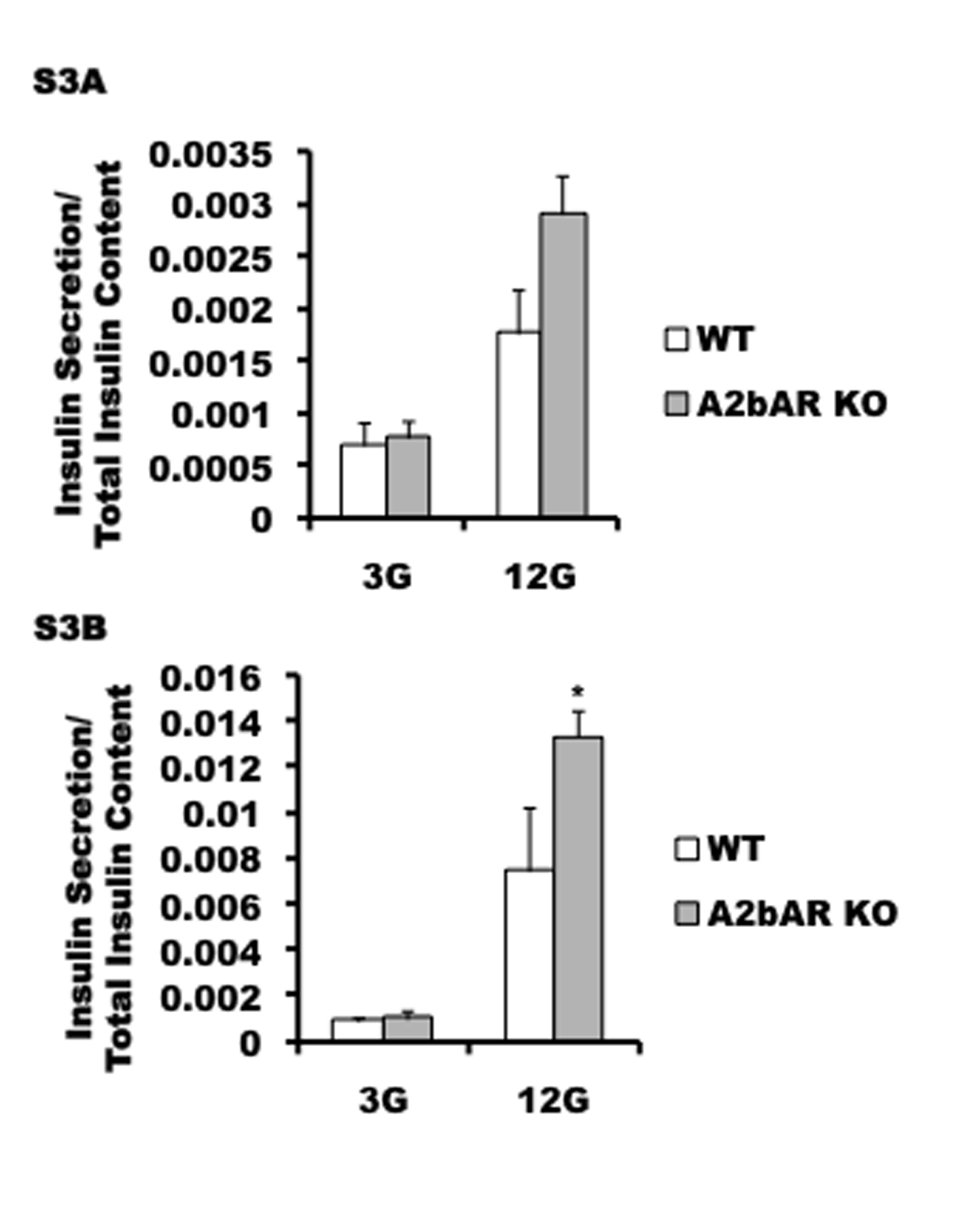

Supplement: Figure S3 — Insulin secretion measured in primary islets. Measurements as described under methods, were carried out in islets treated with basal (3 mmol/L, denoted as 3 G) glucose and high glucose (12 mmol/L, denoted as 12 G) A. at baseline (before HFD) and B. 4 weeks post HFD, p-value = 0.0283. (TIF) [file pone.0040584.s003.tif]

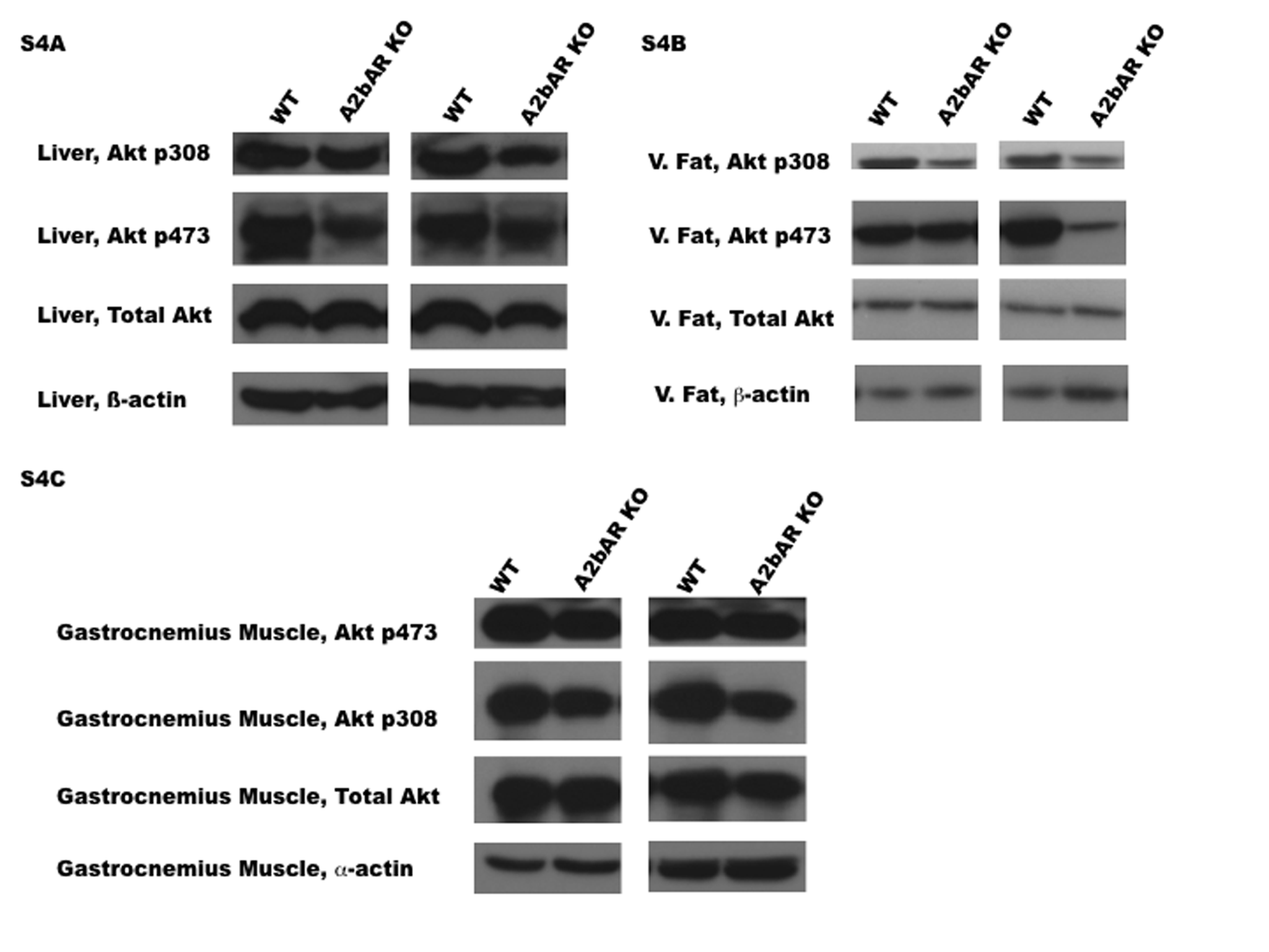

Supplement: Figure S4 — Effect of A2bAR elimination on tissue insulin signaling post HFD. Western blot analysis of additional A2bAR KO and WT pairs for Akt signaling in the liver (A) or fat (B) or gastrocnemious muscle (C), with all details as shown in Figure 2. (TIF) [file pone.0040584.s004.tif]

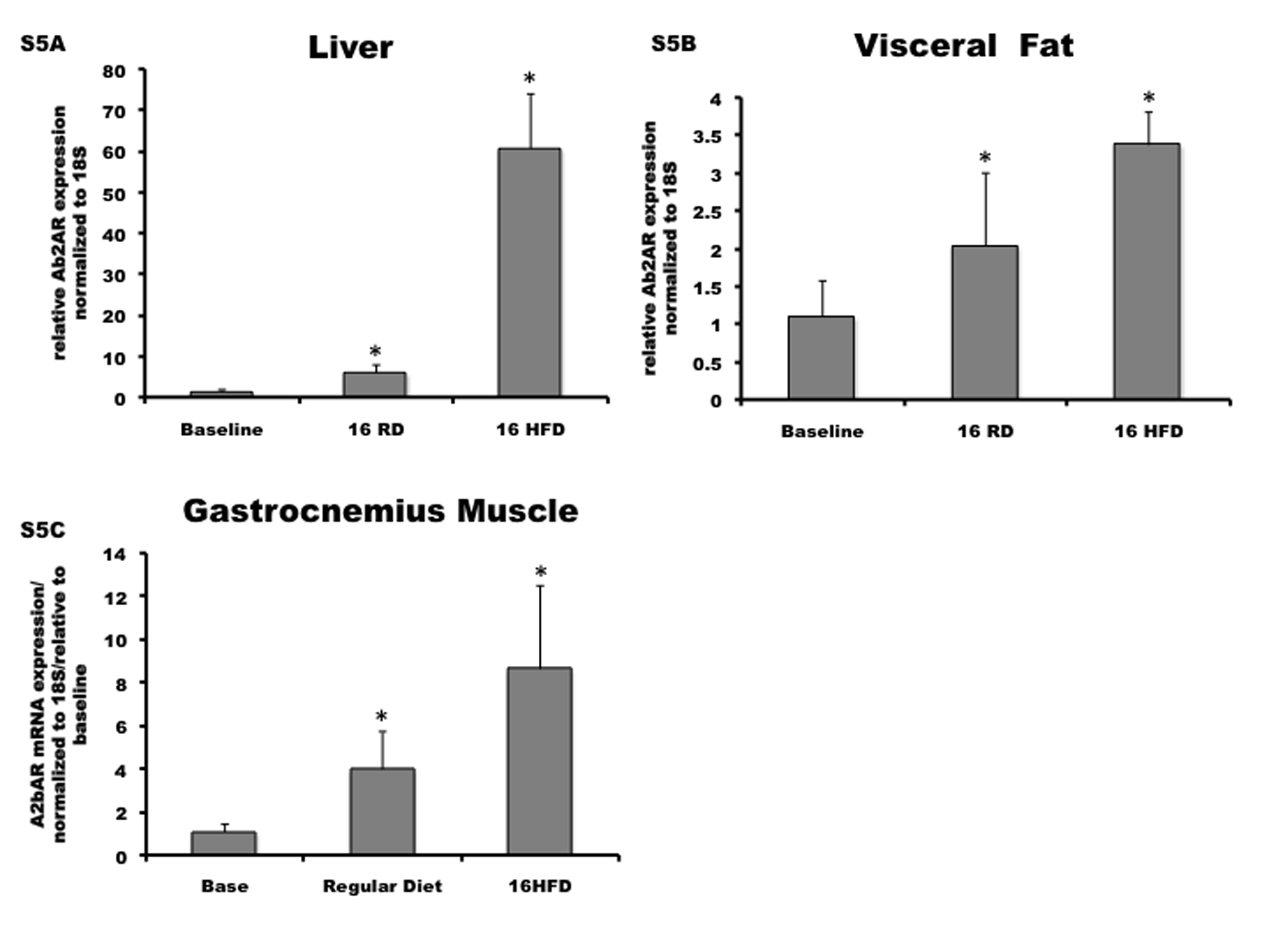

Supplement: Figure S5 — A2bAR tissue expression with age and HFD. Tissue A2bAR mRNA levels in A. Liver, B. Visceral fat, C. Gastrocnemius (Gastroc.) muscle, were measured by qPCR. Twelve-week-old WT mice (C57BL/6J) were analyzed and compared to both similar mice subjected to additional 16 weeks of HFD or an additional 16 weeks of regular chow diet (RD). The expression of the receptor is relative to the baseline value, measured in 12 week old mice before the 16 weeks diet (denoted as HFD or RD), and normalized to 18 S rRNA (denoted as 18 S), p-value = 0.0006, 0.1183×10̂−8 for liver; p-value = 0.0284, 0.1839×10̂−8 for visceral fat; p-value = 0.0011, 0.0009 for gastrocnemious muscle. (TIF) [file pone.0040584.s005.tif]

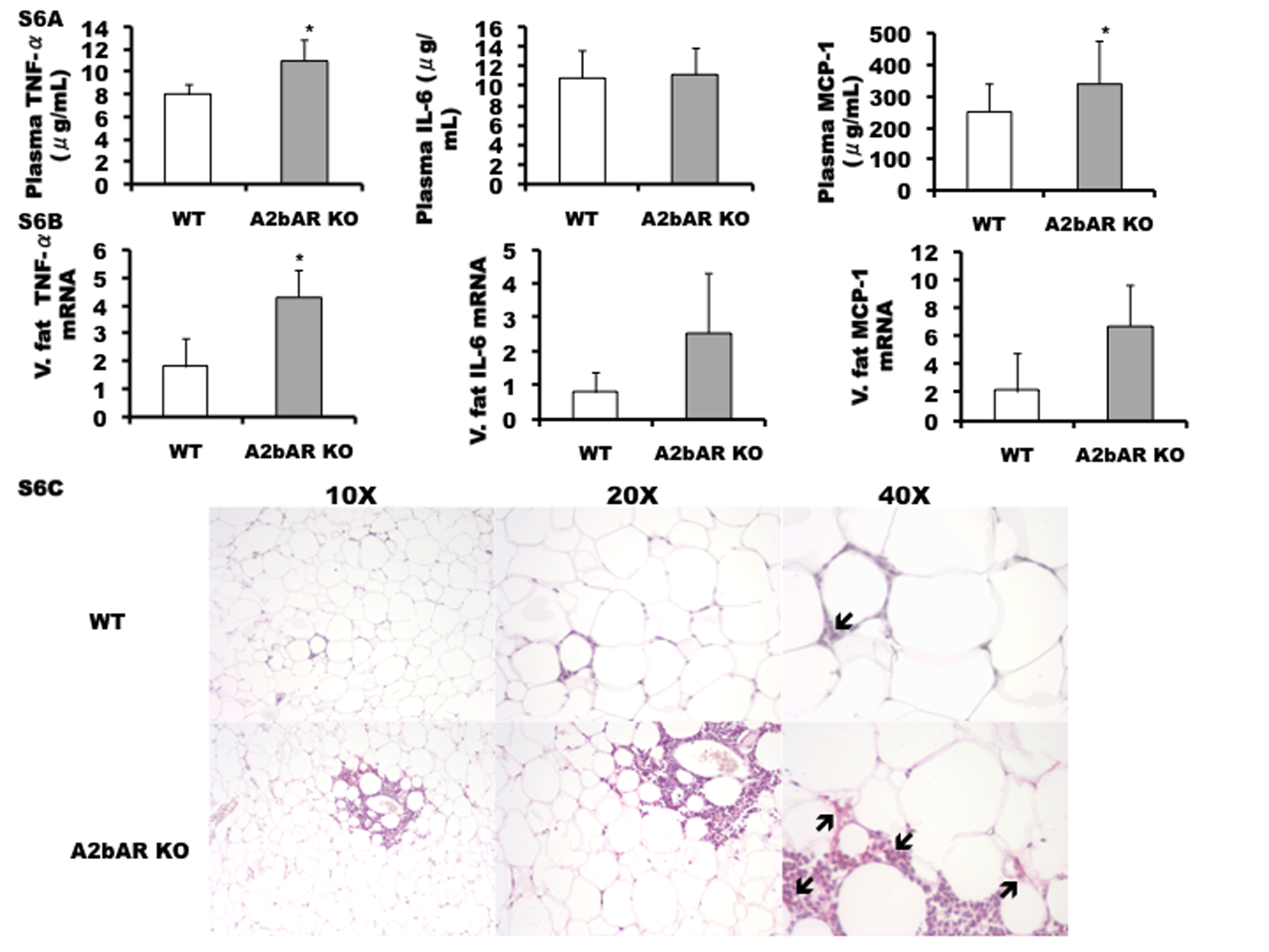

Supplement: Figure S6 — A2bAR elimination causes increase in inflammation and macrophage infiltration. A. TNF-α, IL-6 and MCP-1 levels were measured post 16 weeks of HFD in the plasma, p-value = 0.0007, 0.75116, 0.0392, respectively. The mice were 28 week old upon collection for analysis. B. TNF-α, IL-6 and MCP-1 levels were measured post HFD in the visceral fat, p-value = 0.0367. In each group data is representative of n = 10 for plasma and n = 4 for mRNA levels. qPCR data were normalized to 18 S rRNA and presented as A2bAR KO samples relative to WT (set at 1). C. Morphology of visceral fat paraffin sections (12 week old mice subjected to 16 weeks of HFD, and collected at 28 weeks of age) stained with F4/80 to detect macrophages, and counterstained with hematoxylin, as described in methods. Arrows point to crown structures (F4/80-positive cells). Data are representative of 5 sections and 3 mice per group. There is a trend towards an increase (but not a statistically significant one) in the number of crown structures in the KO samples compared to WT control. Similarly, analysis of fat cell size using Image J software show no statistical difference in adipocyte cell size in WT and KO samples. (TIF) [file pone.0040584.s006.tif]

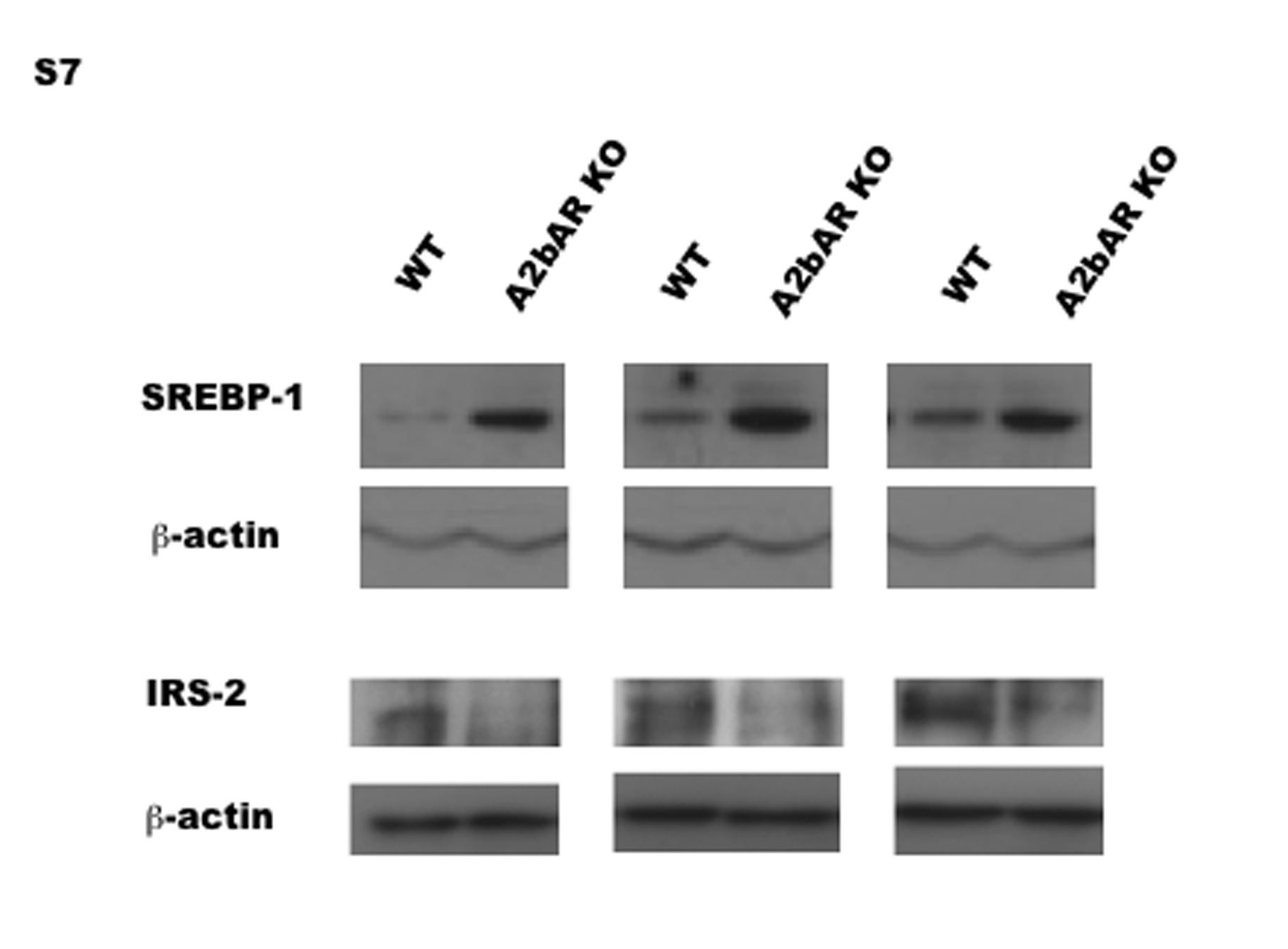

Supplement: Figure S7 — A2bAR elimination causes elevated SREBP-1 and a decrease in IRS-2 levels. Western blot analysis of additional A2bAR KO, WT pairs for SREBP-1 and IRS-2 levels, with all details as shown in Figure 3. (TIF) [file pone.0040584.s007.tif]

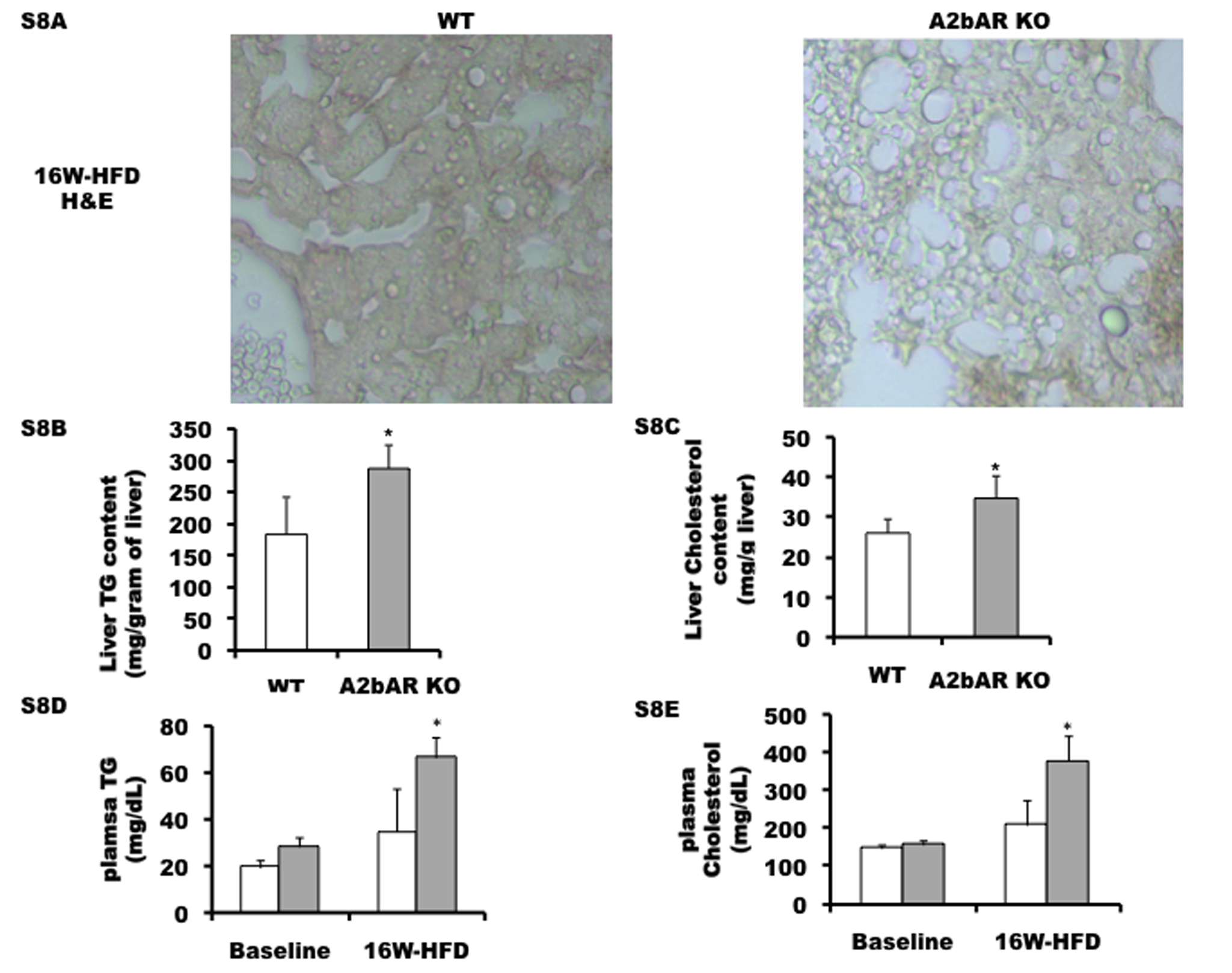

Supplement: Figure S8 — Role of A2bAR in liver lipid homeostasis. A. Liver morphology post 16 weeks HFD depicted in cryo sections stained with H&E as described in the methods. Noted are the fat droplets in the A2bAR KO samples. B., C. Liver cholesterol (n = 6) and triglyceride (n = 6) content in WT and A2bAR KO mice measured as described in Methods. D., E. Plasma lipid levels, including triglycerides (TG), p-value = 0.0002 and cholesterol, p-value = 0.0895×10̂(−3), were measured in 28-week-old male mice following 16 weeks of HFD and 16 hours post starvation. (TIF) [file pone.0040584.s008.tif]

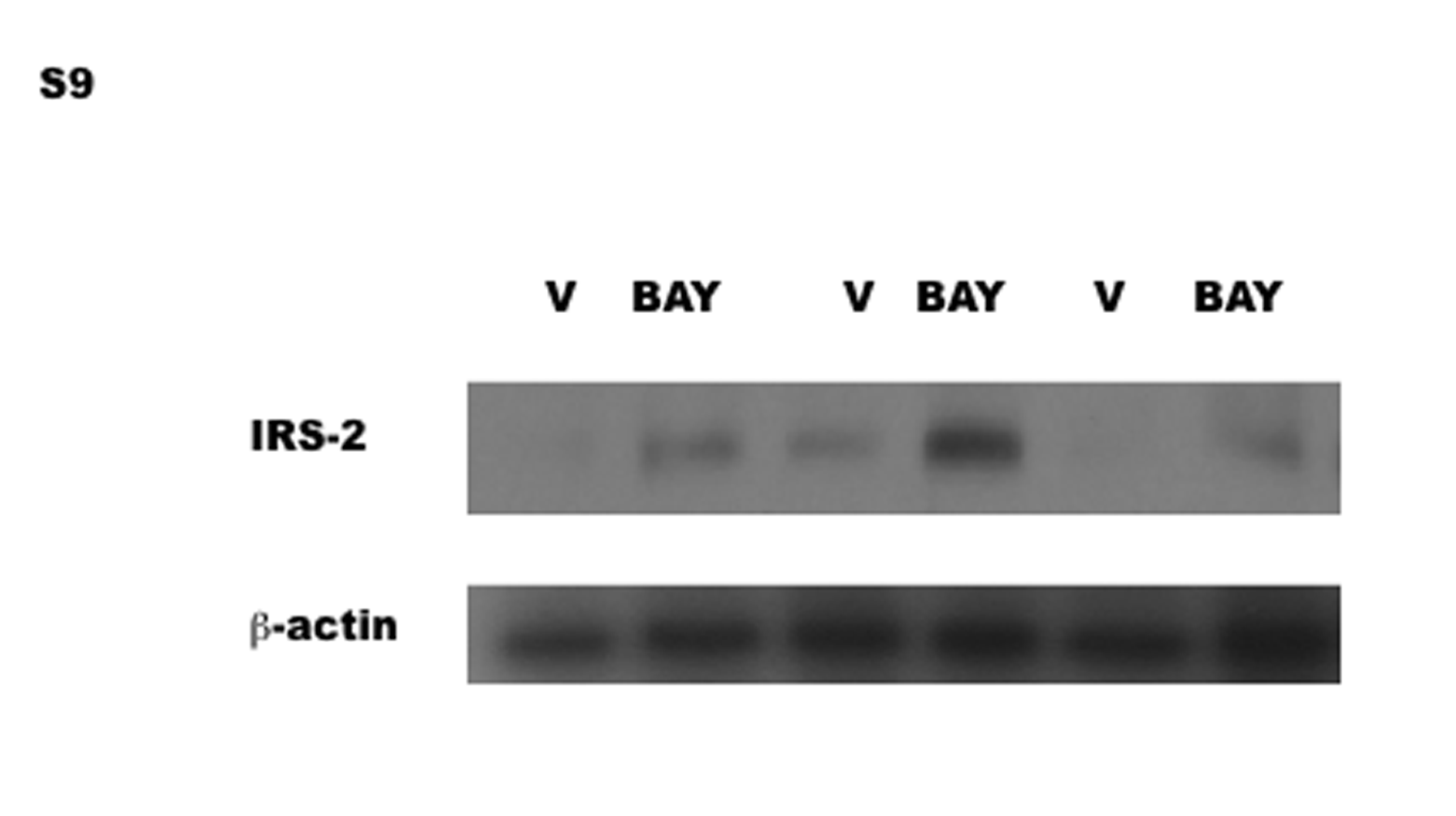

Supplement: Figure S9 — Effect of A2bAR activation on IRS-2 level. Shown are additional analyses of effects of A2bAR activation by vehicle (V) or BAY 60-6583 (BAY) on IRS-2 levels in wild type mice, with all details as shown in Figure 4E. (TIF) [file pone.0040584.s009.tif]

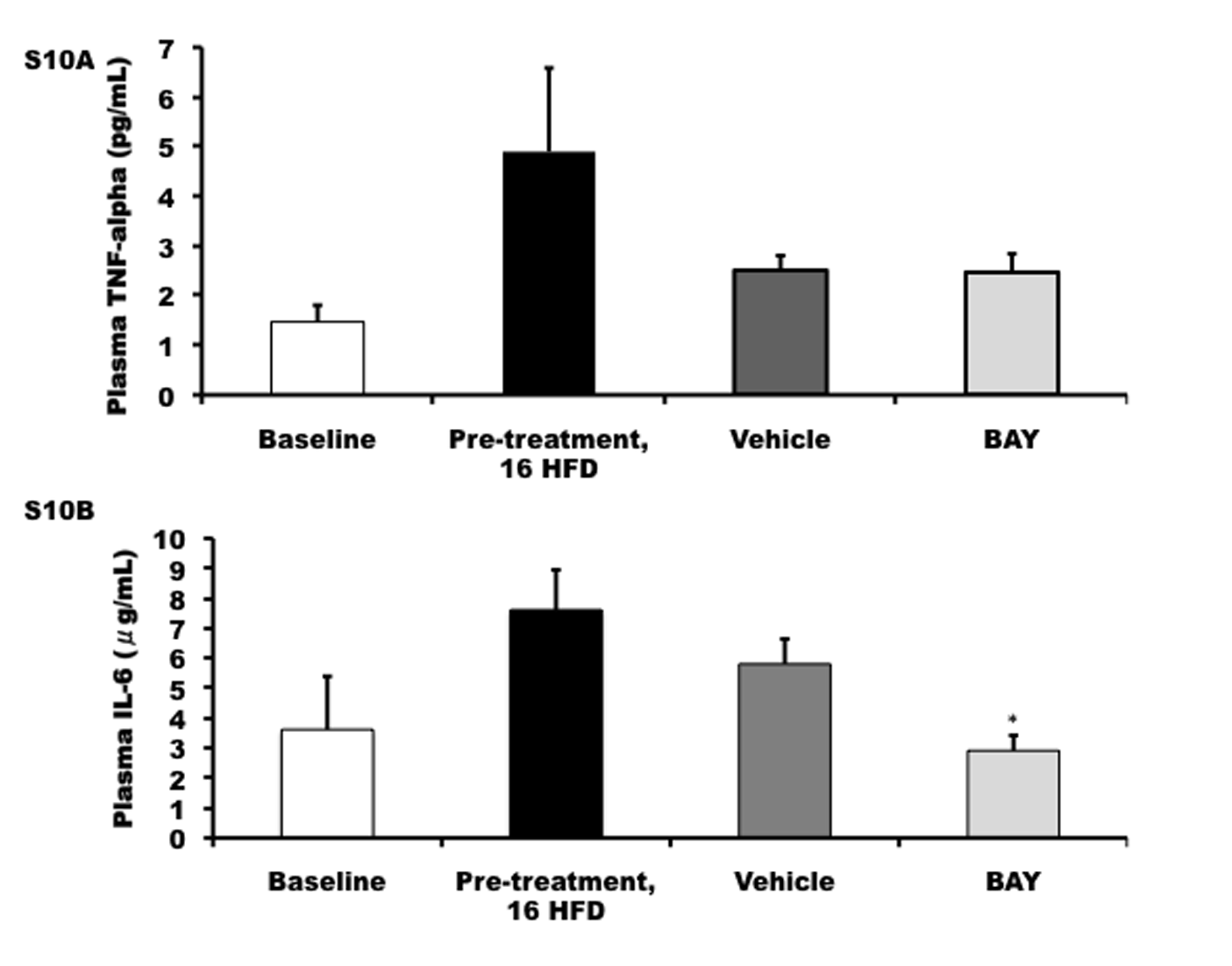

Supplement: Figure S10 — BAY 60-6583 administration ameliorates chronic inflammation. All conditions are as described in the legend to Figure 4. Plasma levels of TNF-α (A) and IL-6 (B), p-value = 0.0138, at Baseline (12 weeks), Pre-treatment (16 weeks HFD), and post-BAY 60-6583 (BAY) or Vehicle injection. (TIF) [file pone.0040584.s010.tif]

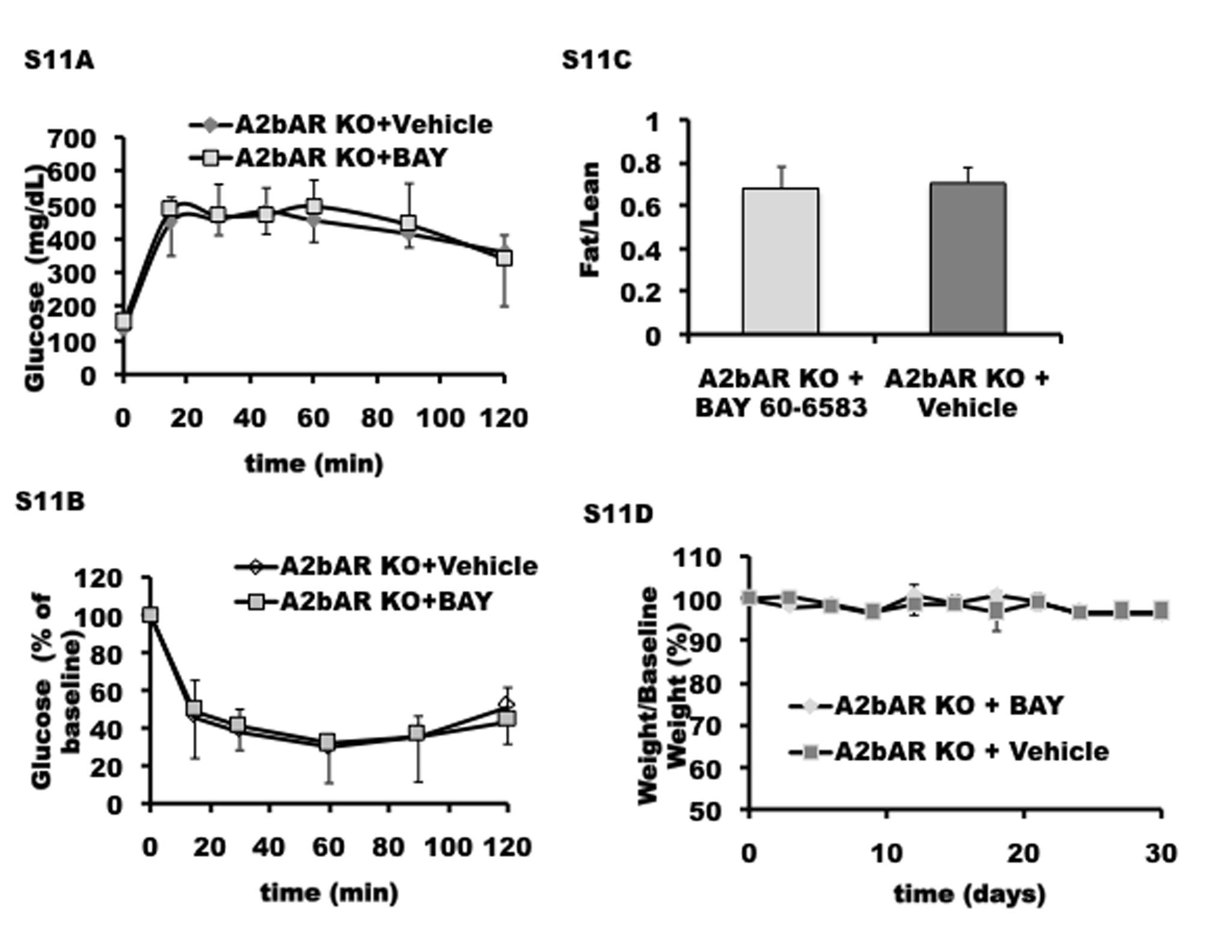

Supplement: Figure S11 — Specificity of BAY 60-6583 in vivo. Glucose parameters were measured in male A2bAR KO (A2bAR KO) 20 W post High fat diet (26 weeks of age), post-4 weeks administration of A2bAR specific agonist BAY 60-6583 (denoted as BAY) or Vehicle. A. Glucose clearance in the blood post glucose overload (n = 5/group), B. Glucose clearance in the plasma post insulin overload (n = 5/group), graphed as percentage of baseline. C. Fat to lean mass ratio (n = 5/group), D. Percent weight gain with 8 weeks BAY 60-6583 treatment (n = 5/group). (TIF) [file pone.0040584.s011.tif]

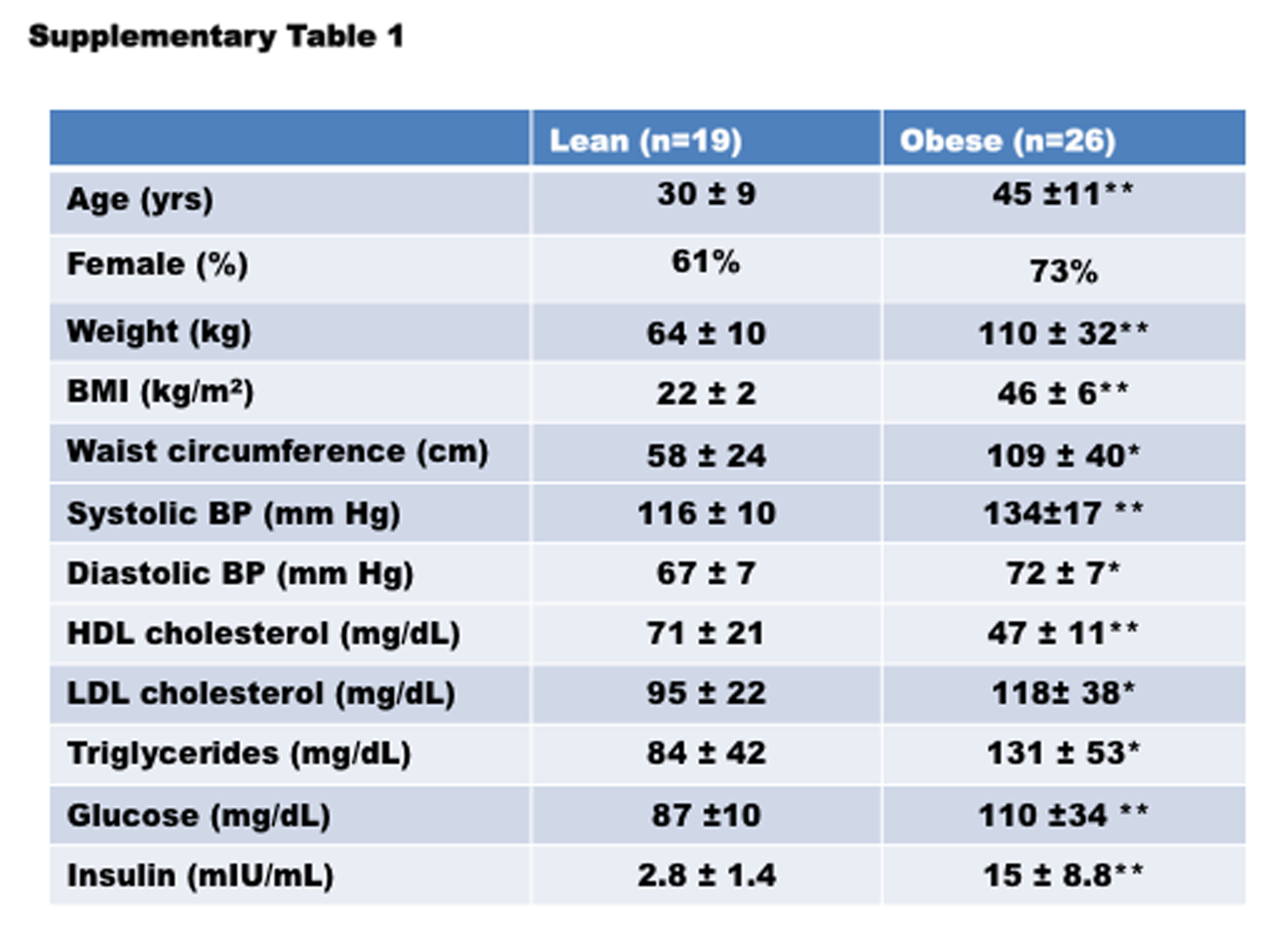

Supplement: Table S1 — Clinical Characteristics. Values are a mean ± standard deviation. The following abbreviations were used: body mass index (BMI), low density lipoprotein (LDL), high density lipoprotein (HDL); * p-value <0.05; **p-value<0.001. (TIF) [file pone.0040584.s012.tif]
