# Supplementary material for: The A2b Adenosine Receptor Modulates Glucose Homeostasis and Obesity
Source: PLoS One. 2012 Jul 25;7(7):e40584. doi: 10.1371/journal.pone.0040584 (PMC3405065; doi:10.1371/journal.pone.0040584)
Supplement: Methods S1 — Supplemental methods are available online labeled as S1 Methods. (DOC) [file pone.0040584.s013.doc]

**Methods S1:**

***Animal models and diets***

The identical background of the experimental groups (C57BL/6J strain) was confirmed by PCR-based gene marker analysis using MAX-BAX (Charles River Laboratories). For this study, we used age-matched male mice, unless specified otherwise. In all experiments, male mice at 12 weeks of age were subjected to either regular chow diet (Teklad, cat# TD2918) or Western diet (Teklad, cat# TD88137) denoted as HFD, for 16 weeks, unless otherwise indicated. Mice were anesthetized by isoflurane. Before tissue collection and plasma analysis, mice were starved for 16 hours. For peripheral tissue insulin signaling mice were injected with 1U/kg Humulin R and 15 minutes before collection of tissues; mice were sacrificed by cervical dislocation and liver, visceral fat pads and gastrocnemius muscle were collected and snap-frozen. All mice procedures were approved and in agreement with the guidelines of and approved by the Institutional Animal Care and Use Committee of the Boston University School of Medicine (Protocol AN14064).

***Oxygen consumption, heat production, respiratory exchange ratio (RER)***

Mice were first acclimated for a period of 24 hours, and then gas exchange, activity, and heat production values were collected periodically up to 48 hours, with the first 24 hours in fed status and the other 24 hours in fasted status. Oxygen consumption and carbon dioxide production were measured for each mouse at 8-minute intervals. Outdoor air reference values were measured after every 10 consecutive O2 and CO2 measurements. Instrument settings were as follows: gas flow rate = 0.5 l/minute; settle time = 90 seconds; measure time = 30 seconds. Gas sensors were calibrated daily with primary gas standards containing known concentrations of O2, and CO2 (Air Gas). A mass flow meter was used to measure and control air flow. Rates of O2 consumption (VO2, ml/h/kg body wt) and of CO2 production (VCO2, ml/h/kg body wt) were measured using electrochemical and spectrophotometric sensors, respectively. Respiratory quotient was calculated as the ratio of VCO2 to VO2. Heat production was determined based on gas exchange according to the manufacturer’s algorithm.

***Body-composition analysis***

The instrument was calibrated prior to each measurement session, using a special phantom provided by the manufacturer. NMR measurements were made by placing live mice into a thin wall plastic cylinder (4.7 cm, inside diameter; 0.15 cm thick), with freedom to turn about but limited to ~4-cm vertical movements by a plastic insert. After 3 minutes, when the measurement was completed, the mice were returned to their home cage. Relative lean mass and fat mass values were calculated by dividing absolute values by body weight.

***Plasma glucose measurements, glucose tolerance test and insulin clearance test***

Mice were put on HFD for 16 weeks, and starved for 16 hours. Tail blood glucose was measured with a Glucometer (One Touch Ultra) before and at 15, 30, 45, 60, 90, 120 minutes post intraperitoneal injection of glucose (1.25 g of glucose per kilogram of body weight dissolved in saline). Insulin clearance was measured in the plasma (cheek bleed) at baseline and post glucose injection (same as above) at 20 and 120 minutes.

***Insulin tolerance test***

Mice were put on HFD for 16 weeks, starved for 6 hours. Tail blood glucose was measured with a Glucometer (One Touch Ultra) before and at 15, 30, 60, 90, 120 minutes post intraperitoneal injection of insulin (1U/kg mouse, Humulin R U-100, Lilly, diluted in saline with Ph7.4).

***Pyruvate tolerance test***

Mice were put on HFD for 16 weeks, starved for 16 hours. Tail blood glucose was measured with a Glucometer (One Touch Ultra) before and at 15, 30, 60, 90, 120 minutes post intraperitoneal injection of sodium pyruvate (Sigma, 2g/kg mouse, dissolved in saline).

***Pancreatic islet isolation and insulin secretion***

Prior to treatments, islets were transferred to islet media with 10% fetal bovine serum and 11mmol glucose for 16 hours. Islets were hand-picked into micro-centrifuge tubes, washed and then incubated in Islet secretion buffer (119 mmol/l NaCl, 20 mmol/l HEPES, 4.6 mmol/l KCl, 1 mmol/l MgSO_4_, 0.15 mmol/l Na_2_HPO_4_, 0.4 mmol/l KH_2_PO_4_, 5 mmol/l NaHCO_3_, 2 mmol/l Ca^2+^, and 0.05% bovine serum albumin, as described in ) at 37°C for 30 minutes, 2 times. They were then incubated with adenosine deaminase (1 U/mL final concentration; Roche Applied Science cat# 10102105001) for 10 min at 37°C. The islets were incubated under the following conditions: basal (3 mmol/L glucose) or high glucose (12 mmol/L glucose) for 30 minutes at 37°C. Insulin secretion was determined by HRTF Insulin Assay (cisbio, 62INSPEB). Insulin secretion data was normalized to total insulin content. These experiments were done with mice at baseline, (12 weeks old), or after 4 weeks of HFD (16 weeks old). This short HFD regime was selected following preliminary experiments.

***Quantitative reverse-transcriptase polymerase chain reaction (qPCR)***

mRNA levels of genes were quantified by using quantitative reverse transcript polymerase chain reaction (qPCR) and the following Applied Biosystems (AB) TaqMan primers: mouse TNF- α (mTNF-α; AB Assay ID # Mm99999068_m1 ); mouse interlukin-6 (mIL-6; AB Assay ID # Mm00446191_m1); mouse monocyte chemotactic protein-1 (mMCP-1; AB Assay ID # Mm_m1); mouse CD11b (mCD11b; AB assay ID # Mm00434455_m1) and levels of the expression each gene were normalized to 18S rRNA (AB part # 4319413E) with the TaqMan Gene Expression Master Mix (AB, part # 4370048), using the ABI 7300 Real-Time PCR System.

***Western Blot Analysis:***

*Liver tissue proteins* were isolated as follows: liver pieces coming from the same lobe (~0.5 cm) were frozen in liquid N_2_, and then homogenized with 500 µl of RIPA buffer (1× PBS, 1% NP-40, 0.5% sodium deoxycholate, 0.1% SDS, 10 mg/ml PMSF, and aprotinin [2 μg/ml]) supplemented with protease inhibitors and the following compounds (0.5 mM NaVa3, 100 nM Ocadeic acid, 50 mM NaF, 5 mM Na-pyro-P04), serving as phosphatase inhibitors. The lysate was vortexed for 10 min at 4 degrees, left on ice for 30 min, and spun down at 16,000 xg for 10 min. The supernatant was carefully removed and used for SDS-PAGE analysis and Western blotting (WB). In each case the supernatant containing the protein layer was gently removed, assayed for protein content by Bradford assay reagent (Biorad, cat# 500-0006), reduced with 2X SDS loading buffer and resolved by 8%-SDS gels (IRS2 total) and 10%-SDS gel for all other proteins (typically, 100μg protein was loaded per lane). Proteins were transferred for 70 min at 100 V on 0.2 µm PVDF membrane (BioRad, cat# 162-0177) with 20% Methanol Transfer Buffer (25 mM Tris, 192 mM Glycine supplemented with 20% MeOH). Membranes post transfers were blocked with 10% milk in 1X PBST (1X PBS, 0.05% Tween), followed by overnight incubation with primary antibody, as follows: dilution of 1:1000 for all that follow: anti-rabbit SREBP1 (Novus Biologicals, cat# NB100-2215), anti-rabbit IRS2 (Cell signaling, cat#3089S), anti-rabbit pAkt(Ser473) (Cell signaling, cat# 4060P), anti-rabbit pAkt(Thr308) (Cell signaling, cat# 2965P), and anti-rabbit Total Akt (Cell Signaling, cat# 4691P). Membranes were washed and incubated for an hour with the appropriate secondary antibodies purchased from Santa Cruz: goat-anti-rabbit (cat# sc2004); goat-anti-mouse (cat# sc2060); donkey-anti-goat (cat# sc2020), at dilutions of 1:10,000, except for β-actin (1:50,000-1:100,000). Proteins were visualized with Chemiluminescent reagent kit (Millipore, cat# WBKLS0500). Blots were then stripped (25 mM Glycine-HCl, 1%SDS, pH 2), reblocked as described above and re-blotted with 1:10,000 dilution anti- mouse β-Actin (Sigma, cat# A5441) to confirm equal loading.

*Muscle tissue proteins* were isolated as follows: small pieces of gastrocnemius muscle (~0.5 cm) were frozen in liquid N_2_, and subsequently ground with 300 µl of RIPA buffer (1× PBS, 1% NP-40, 0.5% sodium deoxycholate, 0.1% SDS, 10 mg/ml PMSF, and aprotinin [2 μg/ml]), supplemented with protease inhibitors and the following compounds (0.5 mM NaVa3, 100 nM Ocadeic acid, 50 mM NaF, 5 mM Na-pyro-P04) serving as phosphatase inhibitors. The lysate was left on ice for 30 min, vortexed for 5 min and spun down at 16,000 xg for 10 min. The supernatant was carefully removed and used for SDS-PAGE analysis and WB. In each case the supernatant containing the protein layer was gently removed, assayed for protein content by Bradford assay reagent (Biorad, cat# 500-0006), reduced with 2X SDS loading buffer and resolved by 10%-SDS gels (for Akt 308, 473, Total Akt, Alpha-Actin) and 8%-SDS gels (for IRS2). Proteins were transferred for 70 min at 100 V on 0.2 µm PVDF membrane (BioRad, cat# 162-0177) with 20% Methanol Transfer Buffer (25 mM Tris, 192 mM Glycine supplemented with 20% MeOH). Membranes post transfers were blocked with 10% milk in 1X PBST (1X PBS, 0.05% Tween), followed by overnight incubation with primary antibody, as follows: dilution of 1:1000 for all that follow: anti-rabbit pAkt (Ser473) (Cell signaling, cat# 4060P), anti-rabbit pAkt (Thr308) (Cell signaling, cat# 2965P) anti-rabbit Total Akt (Cell Signaling, cat# 4691P). Membranes were washed and incubated for an hour with the appropriate secondary antibodies purchased from Santa Cruz: goat-anti-rabbit (cat# sc2004); goat-anti-mouse (cat# sc2060); donkey-anti-goat (cat# sc2020), at dilutions of 1:10,000, except for β-actin (1:50,000-1:100,000). Proteins were visualized with Chemiluminescent reagent kit (Millipore, cat# WBKLS0500). Blots were then stripped (25 mM Glycine-HCl, 1%SDS, pH 2), re-blocked as described above and re-blotted with 1:2000 Alpha Actin specific antibody (Santa Cruz) to confirm equal loading.

*Adipose tissue proteins* were isolated as follows: fat pads of similar size (~0.5 cm) were homogenized after freezing in liquid N_2_, and to each tube 300 µl of RIPA buffer (1× PBS, 1% NP-40, 0.5% sodium deoxycholate, 0.1% SDS, 10 mg/ml PMSF, and aprotinin [2 μg/ml]), supplemented with protease inhibitors and the following compounds (0.5 mM NaVa3, 100 nM Okadaic acid, 50 mM NaF, 5 mM Na-pyro-P04) serving as phosphatase inhibitors. The lysate was left on ice for 30 min, vortexed for 5 min and spun down at 16,000 xg for 10 min. The supernatant was carefully removed and used for SDS-PAGE analysis and WB. In each case the supernatant containing the protein layer was gently removed, assayed for protein content by Bradford assay reagent (Biorad, cat# 500-0006), reduced with 2X SDS loading buffer and resolved by 10%-SDS gels. Proteins were transferred for 70 min at 100 V on 0.2 µm PVDF membrane (BioRad, cat# 162-0177) with 20% Methanol Transfer Buffer (25 mM Tris, 192 mM Glycine supplemented with 20% MeOH). Membranes post transfers were blocked with 10% milk in 1X PBST (1X PBS, 0.05% Tween), followed by overnight incubation with primary antibody, as follows: dilution of 1:1000 for all that follow: anti-rabbit pAkt(Ser473) (Cell signaling, cat# 4060P), anti-rabbit pAkt(Thr308) (Cell signaling, cat# 2965P) anti-rabbit Total Akt (Cell Signaling, cat# 4691P). Membranes were washed and incubated for an hour with the appropriate secondary antibodies purchased from Santa Cruz: goat-anti-rabbit (cat# sc2004); goat-anti-mouse (cat# sc2060); donkey-anti-goat (cat# sc2020), at dilutions of 1:10,000, except for β-actin (1:50,000-1:100,000). Proteins were visualized with Chemiluminescent reagent kit (Millipore, cat# WBKLS0500). Blots were then stripped (25 mM Glycine-HCl, 1%SDS, pH 2), reblocked as described above and re-blotted with 1:10,000 dilution anti-mouse β-Actin (Sigma, cat# A5441) to confirm equal loading.

***Immunohistochemistry:***

***F4/80 Staining***

Paraffin sections of visceral fat (fixed and embedded as in ) were deparaffinized and washed in phosphate buffered saline (PBS) for 5 min. Sections were analyzed with F4/80 macrophage antibody as described previously . The slides were then counterstained with Hematoxylin for 30 sec, followed by wash with continuously running H_2_O until the color under the microscope was not too blue. Slides were then stained with Hematoxylin for 5 dips, followed by 5 dips in each 95 % ethanol/95 % ethanol, 10 dips in each 100 % ethanol/100 % ethanol and 2 min in each Xylene/Xylene, and sealed with Cytoseal-60. 5 sections per mouse were analyzed for positive F4/80 cells.

***H&E staining***

Paraffin sections of liver (fixed and embedded as in ) deparaffinized and washed in phosphate buffered saline (PBS) for 5 min. The slides were then stained in Hematoxylin for 30 sec, followed by wash with continuously running H_2_O until the color under the microscope was not too blue. Slides were then stained with Eosin for 5 dips, followed by 5 dips in each 95 % ethanol/95 % ethanol, 10 dips in each 100 % ethanol/100 % ethanol and 2 min in each Xylene/Xylene, and sealed with Cytoseal-60.

***ELISA for measurement of cytokine levels***

Plasma cytokines were measured according to manufacturer’s instructions, using the following volumes and kits: 50 µl of plasma for TNF-α (eBioscience, cat # 88-7324), and 20 µl of plasma for IL-6, (eBioscience, cat # 88-7064) and 20 µl of plasma for MCP-1 (eBioscience, cat #88-7391). Insulin levels were measured in 5 µl of plasma using ALPCO Insulin EIA kit (cat # 80-INSMSU-E01. Leptin was measured using a Adipokine Multiplex Immunoassay Kit THREE PLEX (Millipore, cat# MADPK-71K-03), following the manufacturer’s instructions. The multiplex assay was resolved by Luminex 100 IS System, Multiplex Bio-Assay Analyzer.

***Human Samples: Sample collection and cDNA Preparation***

Samples from lean individuals (BMI<25) were obtained from the abdominal subcutaneous adipose tissue by percutaneous needle biopsy technique. Subcutaneous adipose tissue sample was collected from the lower abdominal wall during planned bariatric surgery. Samples were immediately placed in RNAlater (Sigma Aldrich) solution and stored at -80°C. Whole adipose tissue was homogenized using ceramic beads with MagNA Lyser (Roche) and total RNA was isolated using the miRNeasy kit (Qiagen, Germantown, MD). Conversion to cDNA was done using the High Capacity cDNA Reverse Transcription Kit (Applied Biosystems). Clinical parameters, as listed in Supplementary Table 1, were measured and recorded for each individual prior to tissue harvesting, including lipid profile, glucose and insulin levels and more, all determined after fasting. All human studies were done in compliance with our institutional review board. Gene expression was determined using the following Applied Biosystems (AB) TaqMan primers: human IRS-2 (hIRS-2; AB Assay ID #Hs00275843_s1); human A2b AR (hA2bAR; AB Assay ID # Hs00386497_m1); and levels of the expression each gene were normalized to human 18S rRNA (AB part # 4319413E) with the TaqMan Gene Expression Master Mix (AB, part # 4370048), using the ABI 7300 Real-Time PCR System.
